# A study on the accumulation model of the Santos basin in Brazil utilizing the source–reservoir dynamic evaluation method

**DOI:** 10.1038/s41598-024-69756-y

**Published:** 2024-08-20

**Authors:** Kangnan Yan, Yinhui Zuo, Yonggang Zhang, Liu Yang, Xu Pang, Siwen Wang, Weiqiang Li, Xu Song, Yiyu Yao

**Affiliations:** 1 State Key Laboratory of Oil and Gas Geology and Exploitation, Chengdu University of Technology, Chengdu, 610059 China; 2 PetroChina Hangzhou Research Institute of Geology, Hangzhou, 310023 China; 3 State Key Laboratory of Continental Shale Oil, Daqing, 163712 China; 4 Daqing Oilfield Exploration and Development Research Institute, Daqing, 163712 China

**Keywords:** Deep-water petroliferous basin, Santos basin, Source rock thermal evolution, Hydrocarbon charging period, Dynamic accumulation model, Geochemistry, Geology

## Abstract

The exploration potential within deep-water petroliferous basins holds great promise for oil and gas resources. However, the dearth of geochemical and isotopic data poses a formidable challenge in comprehending the intricate hydrocarbon charging processes, thereby impeding the comprehensive understanding of hydrocarbon accumulation mechanisms and models. Consequently, the establishment of robust source–reservoir relationships in deep-water petroliferous basins represents a pivotal challenge that significantly influences the exploration strategies and the comprehension of hydrocarbon enrichment dynamics within such basins. In this study, we introduce a novel approach, termed the “source–reservoir dynamic evaluation method,” tailored to investigate reservoir accumulation models in deep-water petroliferous basins. This method uses basin simulation technology to recover the thermal evolution history and hydrocarbon generation and expulsion history of source rocks, and on this basis delimits the hydrocarbon kitchen range. At the same time, the maturity of source rocks corresponding to crude oil and natural gas in typical reservoirs is calculated. Then, when the thermal evolution degree of source rocks adjacent to the reservoir reaches this maturity, the corresponding geological period is the main charging period of hydrocarbon. As a typical deep-water petroliferous basin, the Santos Basin in Brazil has abundant oil and gas reservoirs under the thick salt rock, but there are still some fundamental problems such as unclear oil–gas accumulation process and model. Therefore, in this paper, the main charging periods of typical hydrocarbon reservoirs are determined based on the internal relationship between the thermal evolution history of the main source rocks and the maturity of crude oil and natural gas, and then the hydrocarbon accumulation process is analyzed and the dynamic accumulation model is established. Finally, the favorable prospecting direction is pointed out. The results show that the oil and gas in the Barra Velha Formation in the Santos basin are mainly derived from the Itapema Formation lacustrine shale source rock, and the source rock is mainly developed in the Eastern Sag of the Central Depression, and its main hydrocarbon generation period is from the deposition period of Florianopolis Formation to the deposition period of Santos Formation. The main hydrocarbon expulsion period was from the deposition period of the Santos Formation to the Early deposition of the Iguape Formation. The oil and gas in the Barra Velha Formation were mainly charged from the Late deposition period of the Santos Formation to the Early deposition period of the Iguape Formation. During this period, the hydrocarbon migrated vertically along the normal fault formed in the rift period to the trap of the adjacent inheritance structural highs and accumulated in the reservoir, which was dominated by the accumulation model of the “lower generation-upper reservoir-salt cap”. Since the Barra Velha Formation has the characteristics of near-source accumulation, based on the hydrocarbon expulsion center and hydrocarbon expulsion intensity of the source rock of the Itapema Formation, the distribution ranges of 85% and 50% Pre-salt accumulation probability in the Santos basin were calculated by using the quantitative analysis model of the hydrocarbon distribution threshold. It is suggested that the next oil and gas exploration should be carried out in the paleo-structural highs and slope of Class I favorable area (the hydrocarbon accumulation probability is more than 85%) and Class II favorable area (the hydrocarbon accumulation probability is 85–50%).

## Introduction

According to incomplete statistics, up to now, approximately 2000 oil and gas fields have been discovered in the deep waters of the world’s oceans^1,2^. Remarkably, out of the 283 substantial oil and gas fields identified globally within the past decade (recoverable reserves greater than 2 × 10^8^ bbl oil equivalent), 127 are situated in deep-water and ultra-deepwater regions^1–7^. Noteworthy examples include the Lula field, with a staggering recoverable reserve estimate of 7.16 × 10^9^ bbl oil equivalent; nestled within the Santos Basin of Brazil^2,7,8^. The Julia field, hosting recoverable reserves amounting to 6.02 × 10^9^ bbl oil equivalent, located within the Gulf of Mexico Basin in the United States^9–11^. Therefore, the global deep-water area has become a hot spot for oil and gas exploration^2,5^. Nonetheless, the challenges in deep-water exploration are underscored by instances of unsuccessful ventures, exemplified by the Peroba block within the Santos Basin^7,12^. The predicament arises from the formidable task of procuring rock samples in deep-water petroliferous basins, resulting in an absence of fossil records essential for characterizing the migration and accumulation processes of hydrocarbon fluids within reservoirs. Consequently, it is difficult to analyze the oil–gas charging process, which is the time and the period of hydrocarbon migration to trap, in deep-water petroliferous basins. As a result, the source–reservoir relationship is unclear, which restricts the accuracy of previous understanding of oil–gas accumulation models and laws, and is not conducive to the selection of zones^13–16^. There is an urgent need to establish a new method for determining hydrocarbon charging time in deep water basins where hydrocarbon fluid fossil record data are scarce.

The Santos Basin in Brazil stands out as the world’s most prolific deep-water continental margin basin concerning oil resources. Its ascendancy to global prominence began with the landmark discovery of the Lula oilfield in 2006, prompting Petrobras to channel its focus towards the exploration and development of pre-salt oil reservoirs within the deep-water expanse of the Santos Basin. This strategic shift led to a series of successive discoveries of substantial oil fields, including Jupiter, Iara, Buzios, and Libra, with recoverable reserves of 2.23 × 10^9^, 2.37 × 10^9^, 3.06 × 10^10^, and 1.40 × 10^10^ bbl oil equivalent, resulted in the Santos Basin becoming one of the hot spots for oil and gas exploration in the world nowadays^2,7,17–19^. Many scholars have undertaken extensive research endeavors pertaining to the basic geological characteristics of the Santos Basin, including the tectonic evolution and sedimentary filling^20–27^. Building upon this foundational knowledge, the oil and gas accumulation conditions such as source, reservoir, cap, migration, trap and preservation of the basin are analyzed macroscopically^28–33^. Furthermore, the process of pre-salt hydrocarbon accumulation in the basin was discussed, the hydrocarbon accumulation model was established, and the law of hydrocarbon accumulation was summarized^18,19,34,35^. However, due to the lack of geochemical and isotopic data, the understanding of the source–reservoir relationship is unclear, and the accuracy of the previous understanding of the pre-salt hydrocarbon accumulation model and accumulation law in the Santos Basin needs to be verified.

The formation of oil and gas reservoirs is the migration and accumulation process of hydrocarbon fluids from source rocks to traps, so the timing of reservoir formation must postdate the onset of hydrocarbon generation^36–44^. Therefore, without geochemical and isotope data, the oil and gas charging time can be limited according to the thermal evolution history of source rocks^45^. There are two sets of lacustrine source rocks in the Santos Basin, the Picarras Formation and the Itapema Formation, due to the lack of direct geochemical evidence of source rocks and crude oil, it is difficult to identify the main source rocks of pre-salt oil and gas reservoirs, which restricts the application of the above method in the Santos Basin.

In light of the aforementioned challenges, this paper introduces a novel approach tailored for the investigation of hydrocarbon accumulation models within deep-water petroliferous basins, termed the “source–reservoir dynamic evaluation method.” This method first compares the geochemical parameters of crude oil and natural gas and source rocks in the pre-salt oil and gas reservoirs in the Santos Basin and clarifies the main source rocks under the salt. Then, combined with the thermal evolution history of source rocks and the maturity of crude oil and natural gas, the oil and gas charging time of typical oil and gas reservoirs is determined, and the oil and gas accumulation process is analyzed. Consequently, a dynamic reservoir accumulation model is established. Ultimately, a favorable exploration zone is pointed out, thereby providing invaluable guidance for the continued exploration and development endeavors within the Santos Basin, as well as other deep-water basins globally.

## Geological setting

**Figure 1:**
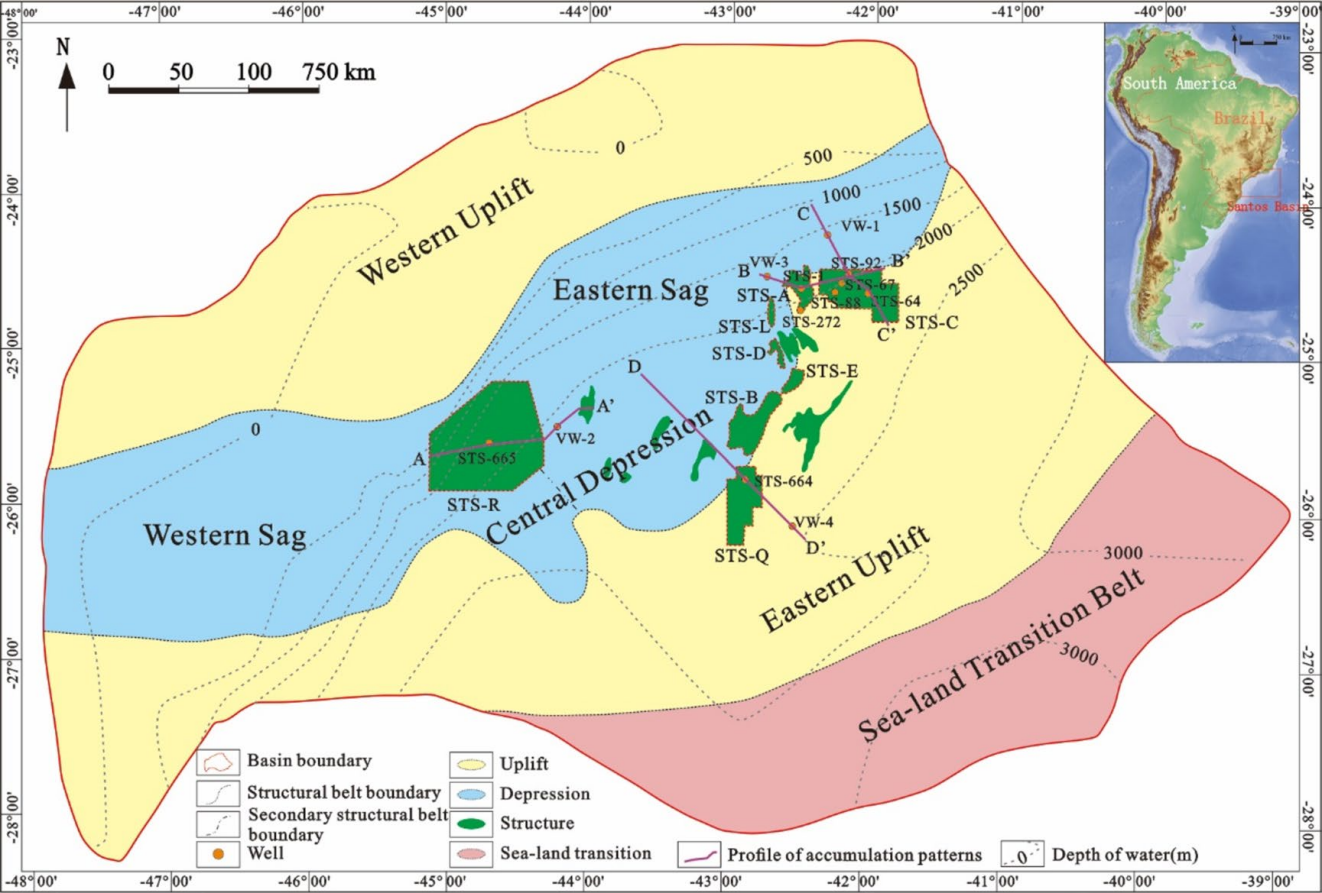
Structural unit division and position of the Santos Basin^45^. Areas with water depths greater than 500 m are considered a deep-water segment. Figures were produced by CorelDRAW Graphics Suite X8 (https://www.corel.com/en/) and 91weitu v19.3.4 (https://www.91weitu.com).

The Santos Basin, the largest sedimentary basin in the Brazilian Sea, is located in the southeastern sea area of Brazil (between latitudes 23° to 28° 30′ S and longitudes 39° 30′ to 48° 30′ W), bounded by the Cabo Frio Arch in the northeast, the Florianopolis Arch in the southwest, the Rio de Janeiro in the northwest, the Upper Cambrian in the west, the Sao Paulo Ridge in the south, and the Charcot Sea Mounts in the southeast. The area is 35.2 × 10^4^ km^2^, and the maximum water depth is more than 4000 m^12,46–48^. The oil and gas fields exhibit an “outer oil, inner gas” in the plane, with crude oil characteristics being “heavier outside, lighter inside,” and the oil and gas reserves indicating “larger outside, smaller inside.” From the perspective of the vertical distribution of oil and gas reservoirs, it can be divided into two sets of accumulation systems: pre-salt and sub-salt. As a whole, the Pre-salt structure is NE-SW trending and has an alternate tectonic pattern of uplift and depression, which can be divided into the Western Uplift zone, the Central Depression zone, the Eastern Uplift zone, and the Sea-land Transition belt from west to east, among which the Central Depression zone can be further divided into the Western Sag and the Eastern Sag (Fig. 1)^45^.

The Santos Basin is a prototypical passive continental margin basin, and its tectonic evolution is closely related to the Atlantic rifting. It mainly experienced three stages of tectonic evolution: the rift stage, the transition stage, and the drift stage^34,49^.

**Figure 2:**
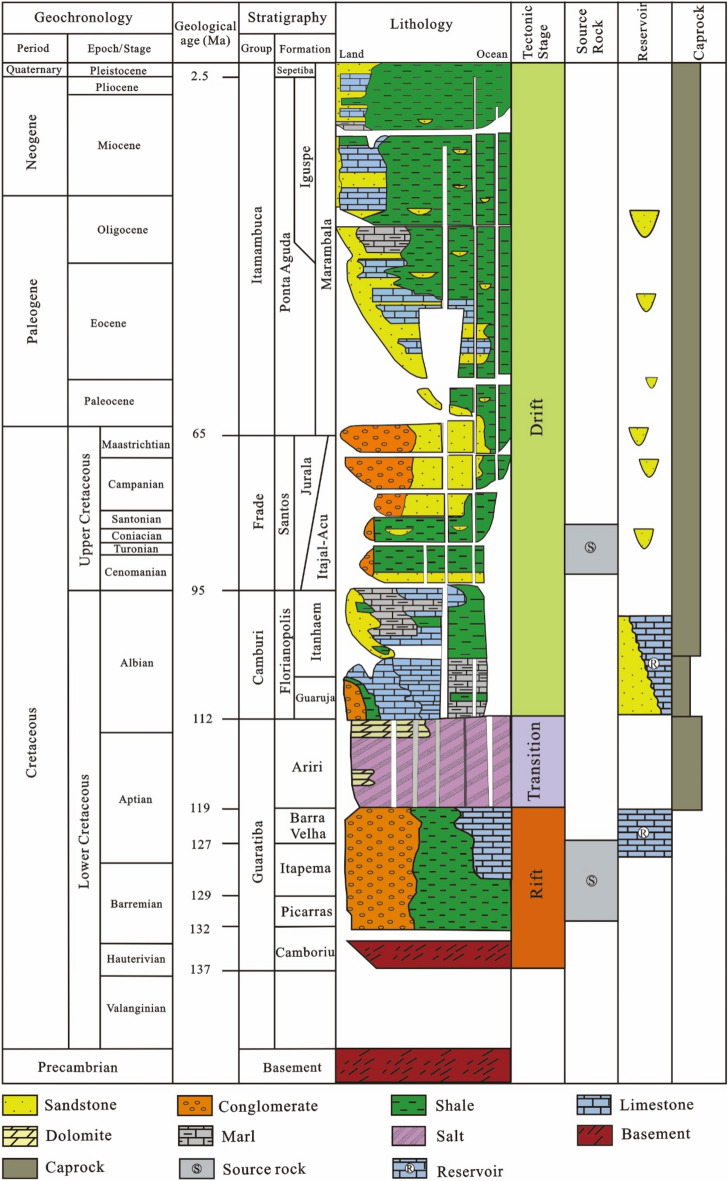
Stratigraphic chart of the Santos Basin^45^.

During the rift stage, the upwelling of mantle material induced crustal uplift, simultaneously causing the South American continent and the Atlantic Ocean to split. The Santos Basin at the top of the dome began to undergo rifting. Above the Precambrian crystalline basement, the Lower Cretaceous Camboriu Formation, a lacustrine sandy mudstone and limestone of lacustrine mega sequence, has successively deposited from bottom to top, comprising the basement volcanic rocks, the Early Cretaceous Picarras Formation, the Itapema Formation, and the Barra Velha Formation. Crucially, the Picarras Formation and Itapema Formation lacustrine shale are important source rocks of pre-salt, while Barra Velha Formation develops algal stromatolite limestone and spherulite limestone, which is the main reservoir of pre-salt. During the transitional stage, intermittent incursions of seawater into the central South Atlantic basin occurred, with a sluggish subsidence rate characterizing the basin. The lake water further salinized to form a set of unevenly distributed and wide-ranging Ariri Formation evaporite, which was the regional cap rock. During the drift stage, the expansion of the mid-Atlantic ridge located between Brazil and Africa led to crustal cooling and shortening. Within this phase, a sequence of marine mega-sequences evolved. From bottom to top, including the Florianopolis Formation sandstone, the Guaruja Formation carbonate rock, the Itanhaem Formation gray mudstone, the Itajai-Acu Formation, the Santos Formation and the Jureia Formation mudstone and turbidite sandstone, and the Marambaia Formation, the Iguape Formation, the Ponta Aguda Formation and the Sepetiba Formation turbidite sandstone, mudstone and carbonate rock were successively deposited (Fig. 2)^30,45^.

## Methods and parameters

### Source–reservoir dynamic evaluation method

Considering the unique challenge posed by the absence of geochemical and isotopic data necessary for characterizing the migration and accumulation of hydrocarbon fluids within reservoirs in deep-water petroliferous basins, we introduce a novel approach, the “source–reservoir dynamic evaluation method.” This method combines basin simulation technology and hydrocarbon maturity to restore the hydrocarbon charging period and is suitable for studying the hydrocarbon accumulation model in deep-water petroliferous basins.

**Figure 3:**
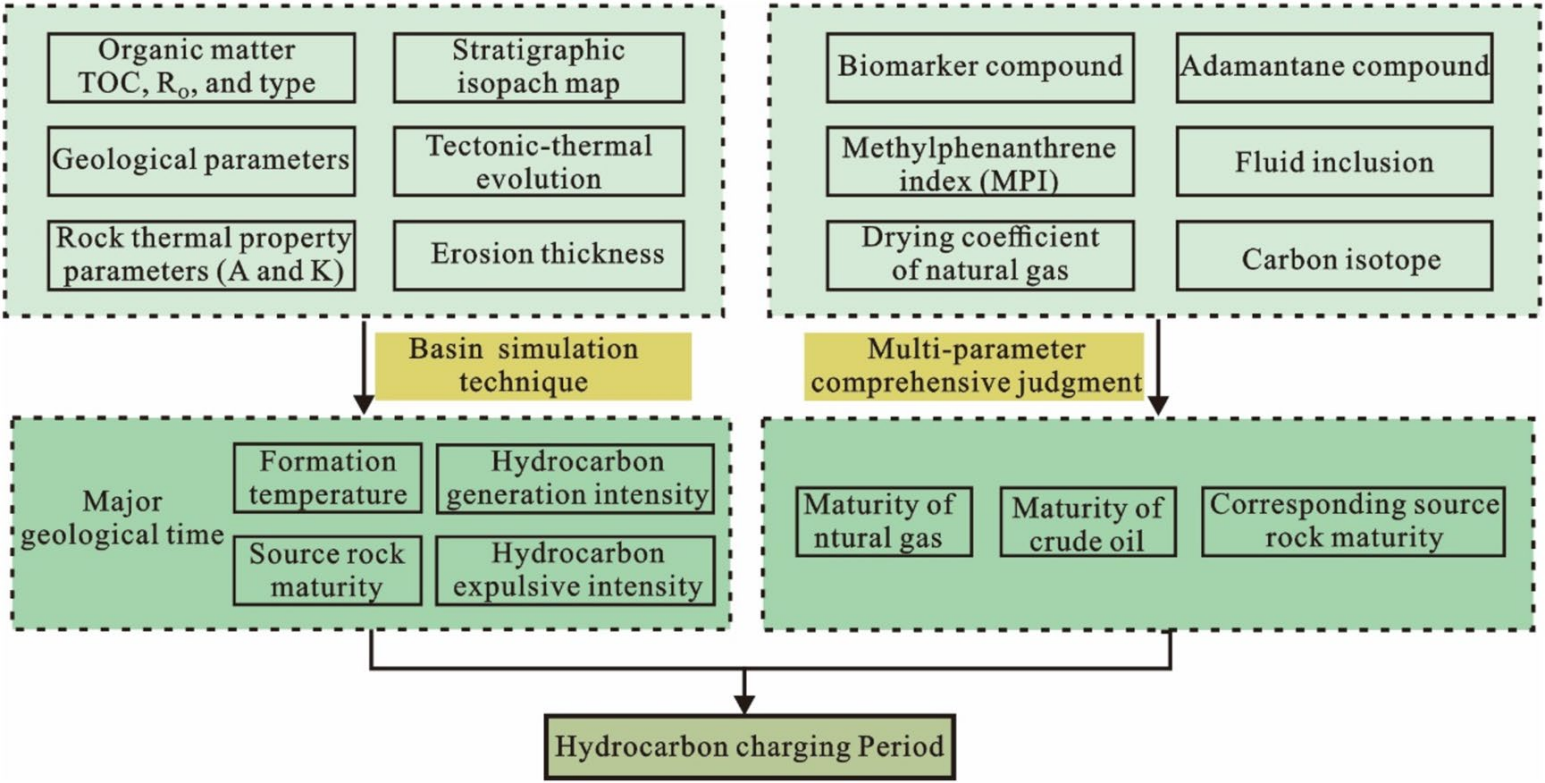
Oil and gas charging period analysis flow chart. TOC = Total Organic Carbon; R_o_ = Vitrinite Reflectance; A = Heat Productivity; K = Coefficient of Thermal Conductivity.

This method first needs to clarify the source rock formations through oil/gas-source rock correlation. Subsequently, advanced basin simulation technology, Basinview and BasinFlow, is employed to meticulously reconstruct vital parameters, including the formation temperature, maturity, hydrocarbon generation intensity, and hydrocarbon expulsion intensity within the source rock strata in the main geological periods, and the source kitchen was delineated according to the hydrocarbon generation and expulsion intensity. On the other hand, the maturity of crude oil and natural gas is calculated respectively using geochemical parameters, and the maturity of source rocks corresponding to crude oil and natural gas in the reservoir is calculated. The oil–gas charging period is the geological period when the thermal evolution degree of the adjacent hydrocarbon kitchen reaches the maturity of source rocks corresponding to crude oil and natural gas. Finally, a virtual well is established in the source kitchen, and the main charging period of oil and gas is analyzed by simulating the hydrocarbon generation and expulsion history of the source rock using software BasinMod 1D within the virtual well (Fig. 3).

To ascertain the reliability of the source–reservoir dynamic evaluation method, a validation process was conducted in the STS-C block of the Santos Basin. The traditional fluid inclusions were used to analyze the oil and gas charging period in the STS-C block, and compared with the main oil and gas charging period determined by the thermal evolution history of the source rock and the maturity of crude oil and natural gas. Should the results obtained via these two distinct methodologies exhibit a high degree of congruence, it would substantiate the credibility and reliability of the novel method introduced within this paper.

### Basic parameters

The successful implementation of the source–reservoir dynamic evaluation technique relies on several key parameters and data types, including: (a) the organic geochemical parameters of source rocks, such as organic matter abundance, organic matter type, and organic matter maturity; (b) the basic geological parameters were obtained by combining seismic and logging data, such as stratigraphic thickness map, denudation amount, lithology, and stratigraphic thermal parameters (A, K); (c) crude oil geochemical parameters, such as biomarker compounds, adamantane compounds, hydrocarbon inclusions, and methylphenanthrene index^50^; (d) natural gas geochemical parameters, such as drying coefficient and carbon isotope^51,52^ (Fig. 3). The geochemical data for the crude oil and natural gas are derived from measured data from the Wells drilled in each block. Due to the lack of actual drilling in the depression area where the source rocks are mainly developed, the geochemical characteristics of well STS-88 in block STS-C were referred to in the process of establishing the virtual well.

Among them, the conversion relationship between the methylphenanthrene index and crude oil maturity is as follows^50^:

1 $$ {\text{R}}_{{\text{o}}} = 0.{6}\;{\text{MPI}}_{1} + 0.{4},\;0.{65}\% < {\text{R}}_{{\text{o}}} < {1}.{35}\% $$

2 $$ {\text{R}}_{{\text{o}}} = - 0.{6}\;{\text{MPI}} _{1} + {2}.{3},\;{1}.{35}\% < {\text{R}}_{{\text{o}}} < {2}.00\% $$

3 $$ {\text{MPI}} = \frac{{{1}{\text{.5}} \times {\text{[(3 - methylphenanthrene}} +{\text{2 - methylphenanthrene)]}}}}{{{\text{[(Phenanthrene}} + {\text{9 - methylphenanthrene}} + {\text{1 - methylphenanthrene)]}}}} $$

where R_o_ is the maturity of crude oil, %; MPI is the methylphenanthrene index.

## Results

### Oil/gas-source rock correlation

#### Characteristics of crude oil biomarker compounds

**Figure 4:**
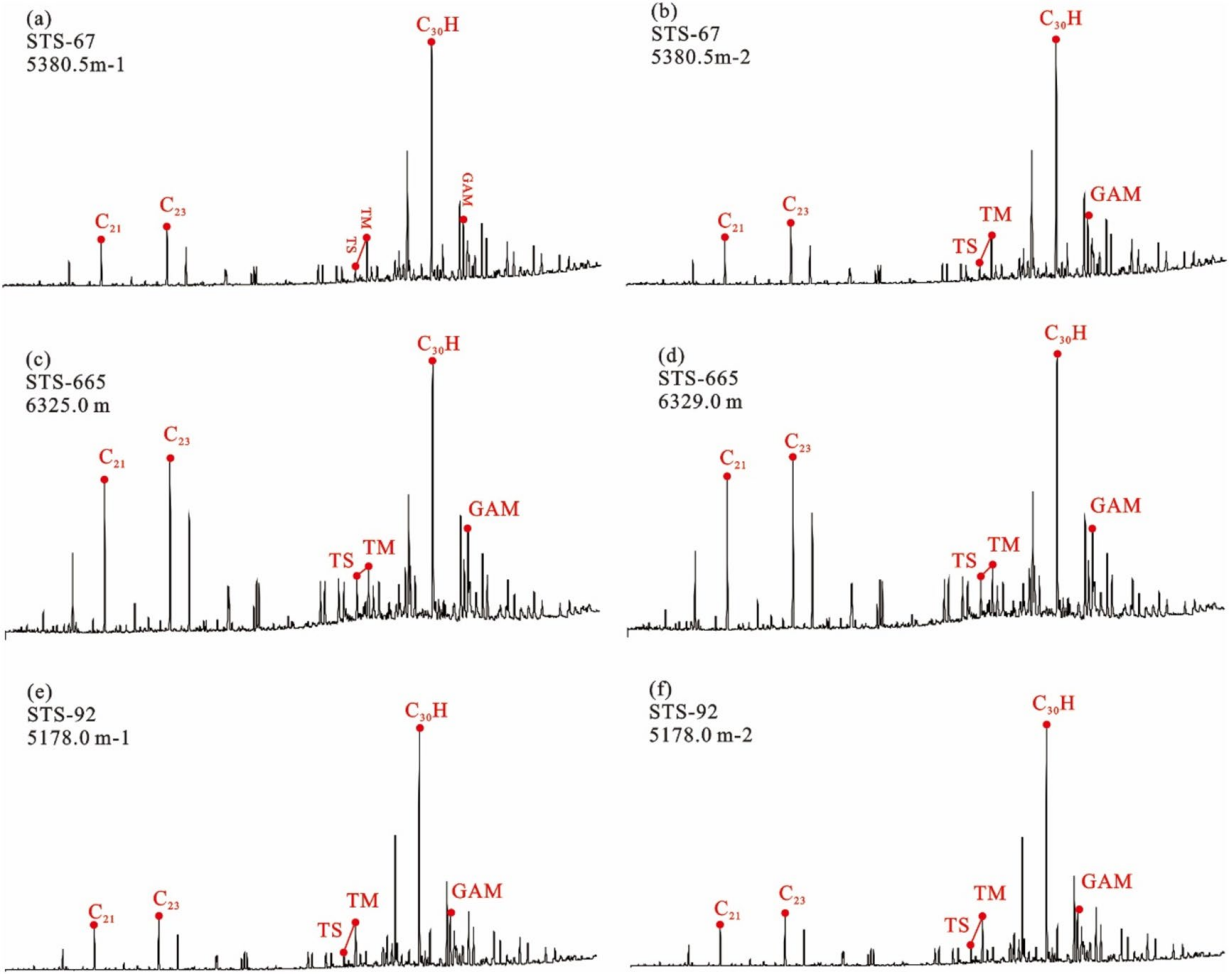
Distributions of tricyclic terpanes (m/z 191) in the representative crude oil samples from the Barra Velha reservoir.

**Figure 5:**
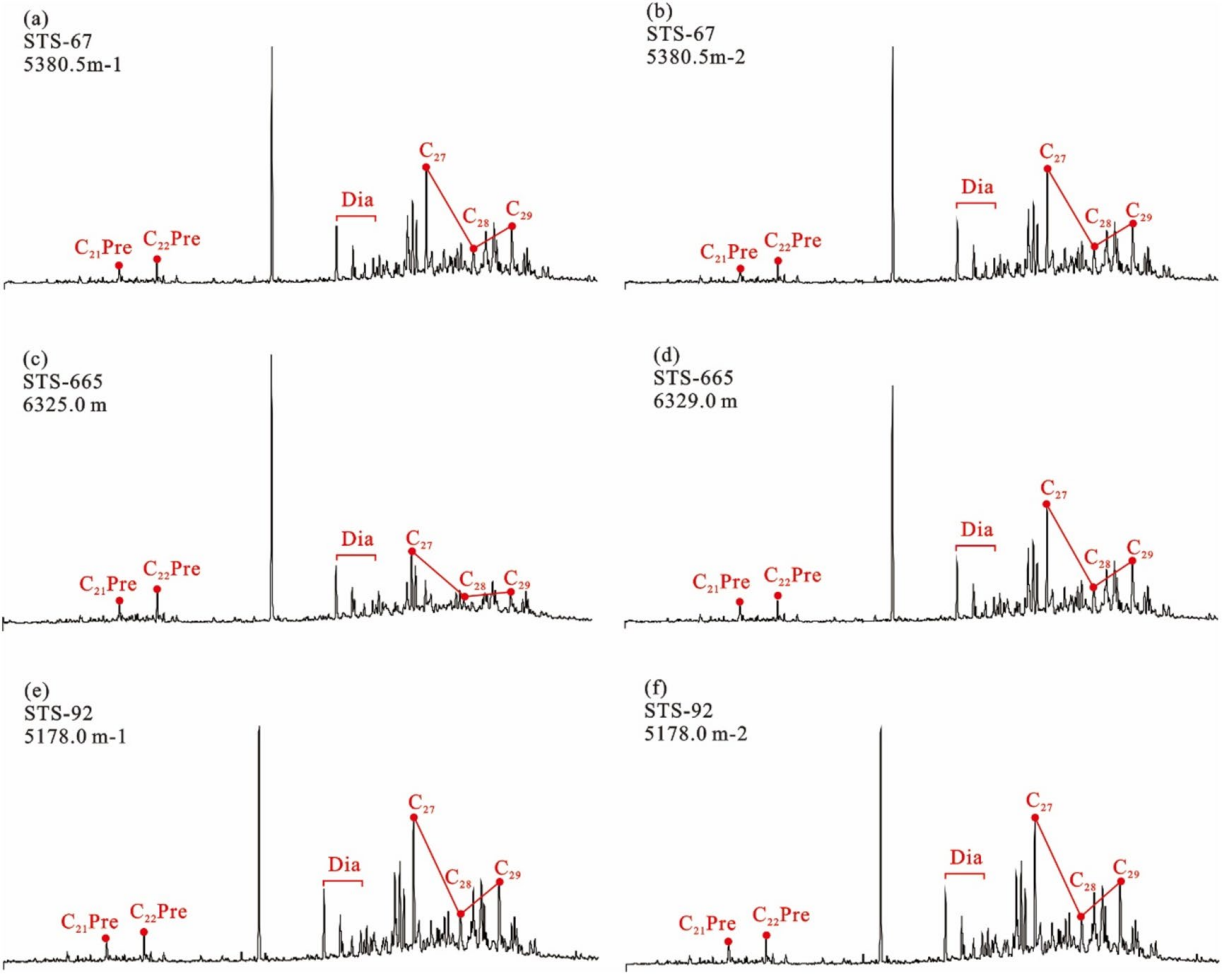
Distributions of regular steranes (m/z 217) in the representative crude oil samples from the Barra Velha reservoir.

The long-chain tricyclic terpene + hopane series (m/z 191) Gas chromatography–mass spectrometry (GC–MS) analysis showed that the tricyclic terpene of crude oil from Barra Velha Formation in Santos Basin was mainly C_23_, Ts (trisnorneohopane) < Tm (trisnorhopane), and the content of gammacerane was higher (Fig. 4). The sterane series (m/z 217) showed that the content of rearranged sterane series in the sterane series of crude oil in the Barra Velha Formation was high, and the regular sterane was dominated by C_27_ sterane, showing an asymmetric V-type distribution (Fig. 5).

The 20S/(20S + 20R) ratios and the ββ/(αα + ββ) ratios, C_29_ sterane parameters, are commonly used and reliable maturity parameters with a wide application range, and their values increase with the increase of thermal evolution degree. The C_29_ 20S/(20S + 20R) ratios of saturated hydrocarbon C_29_ sterane in crude oil is between 0.372 and 0.51, indicating that the isomerization degree of sterane in crude oil is high, and the maturity of crude oil is in the middle-high maturity stage (Fig. 5).

#### Characteristics of natural gas compositions and carbon isotopes

**Figure 6:**
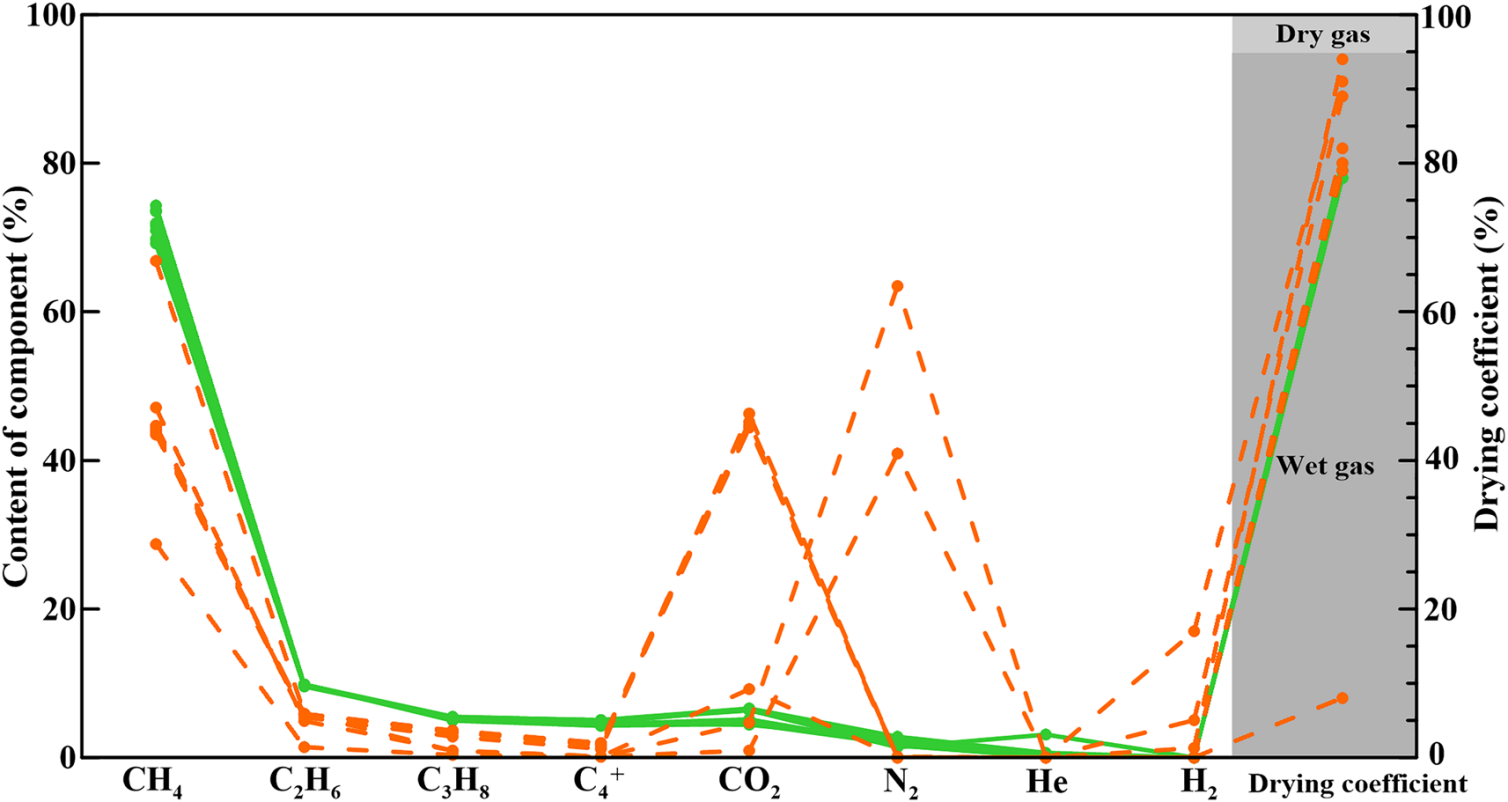
Natural gas components and drying coefficient of Barra Velha Formation, Santos Basin (The green dots represent the STS-R block, the orange dots represent the STS-C block).

The methane content of natural gas in STS-R block and STS-C block of Barra Velha group in the study area is between 44.22 and 74.30%, the ethane content is between 1.40 and 9.84%, the propane content is between 0.35 and 5.04%, and the drying coefficient is between 0.78 and 0.91. It is characterized by wet gas and low maturity. The content of CO_2_ is between 0.94 and 46.29%, and the content of the STS-C block is higher than that of the STS-R block (Fig. 6).

**Figure 7:**
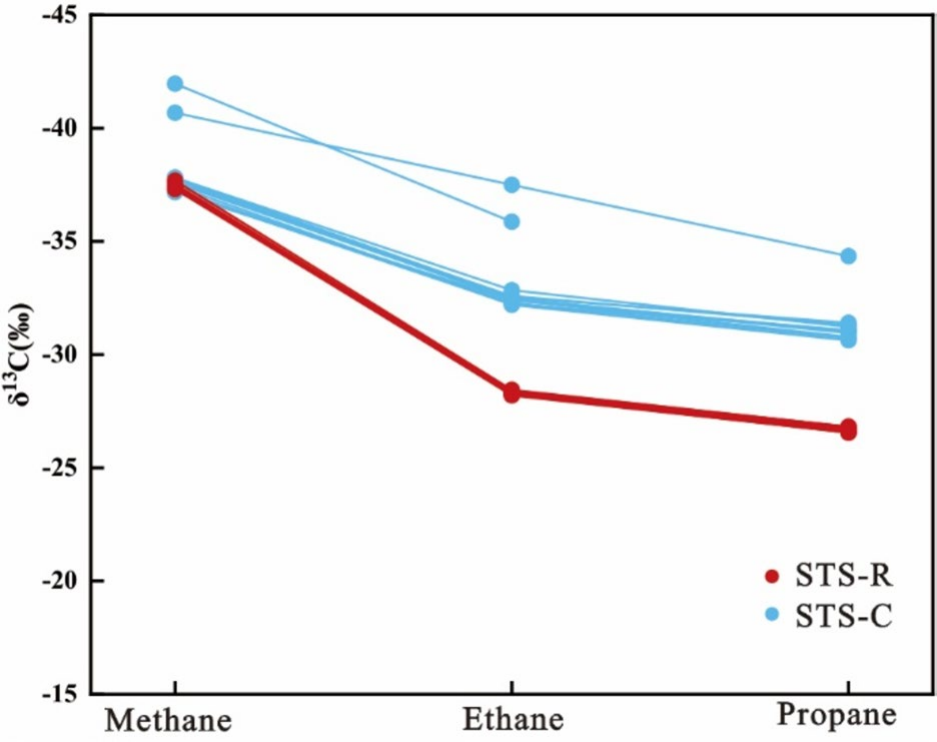
Carbon isotope sequence of natural gas from Barra Velha formation, Santos Basin.

The carbon isotopic composition of methane in natural gas of STS-C block is between − 37.02 and − 45.14‰ (average value is − 38.63‰). At the same time, the ethane is between − 32.10 and − 38.88‰ (average value is − 33.38‰), and the propane is between − 30.54 and − 31.40‰ (average value is − 30.76‰) (Fig. 7).

The carbon isotopic composition of methane in natural gas of STS-R block is between − 37.35 and − 45.28‰ (average value is − 39.14‰). At the same time, the ethane is between − 28.20 and − 28.44‰ (average value is − 28.31‰), and the propane is between − 26.55 and − 26.81‰ (average value is − 26.67‰) (Fig. 7).

#### Characteristics of source rock biomarker compounds

**Figure 8:**
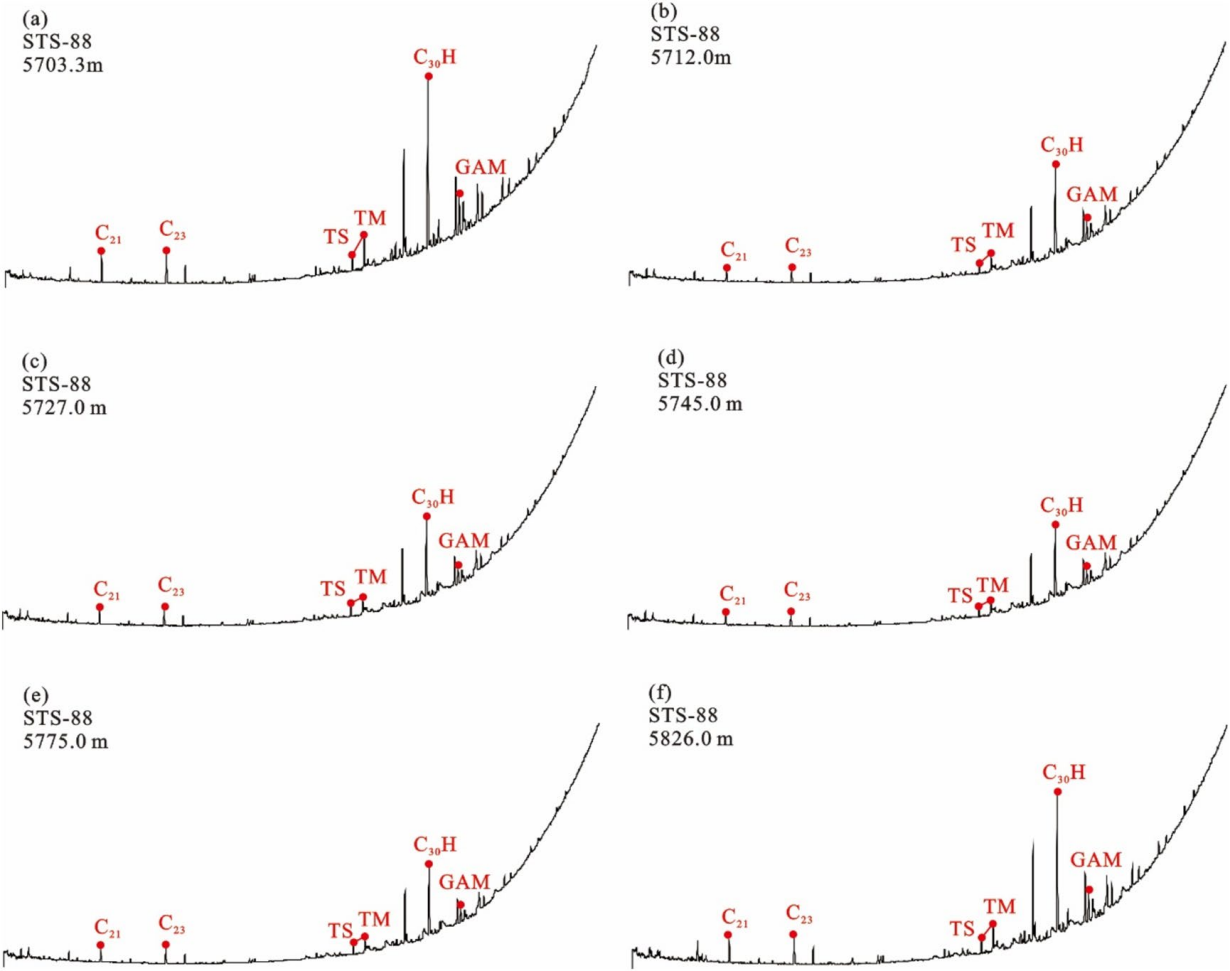
Distribution of terpanes in the source rocks from the Itapema Formation in Santos basin (m/z 191).

**Figure 9:**
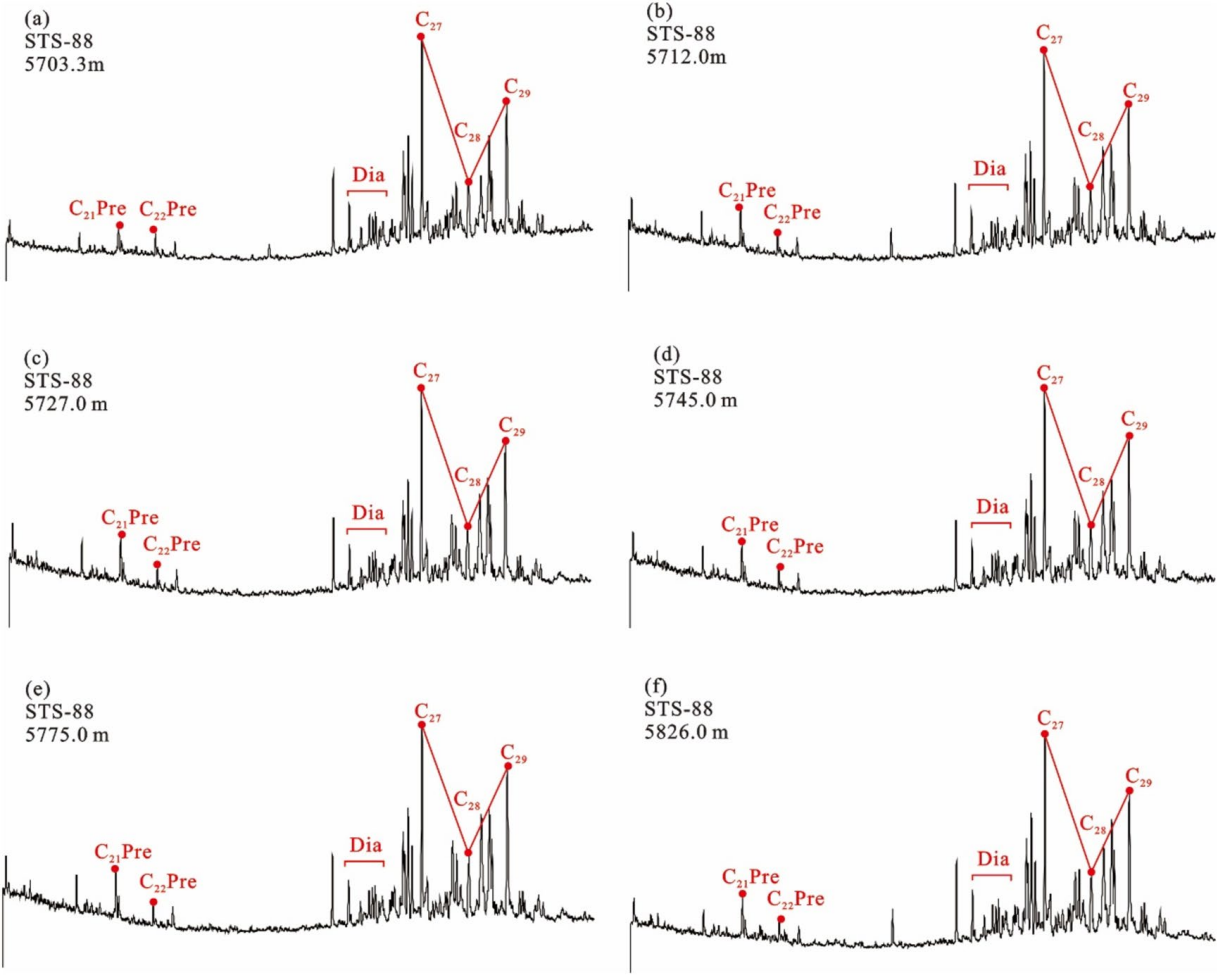
Distribution of regular steranes in the source rocks from the Itapema Formation in Santos basin (m/z 217).

The tricyclic terpene of the Itapema Formation source rock in the Santos Basin is dominated by C_23_, Ts < Tm, and the content of gammacerane is high (Fig. 8). The content of rearranged steranes in the sterane series of the Itapema Formation source rocks is high, and the regular steranes are dominated by C_27_ steranes, showing an asymmetric V-type distribution. The 20S/(20R + 20S) value of saturated hydrocarbon C_29_ steranes indicates that the isomerization degree of steranes in source rocks is high (Fig. 9).

#### Result of oil/gas-source rock correlation

Due to the high similarity of biomarker compounds of crude oil in the STS-C block and STS-R block, it can be considered that the genetic relationship between the source rocks of these two blocks is better (Fig. 4). In addition, the carbon isotopic composition sequence of natural gas in the STS-C block and STS-R block has not been reversed, indicating that they are all from a single natural gas source (Fig. 7). These findings collectively support the hypothesis that the Pre-salt oil and gas resources within the study area, including both the STS-C block and STS-R block, are likely derived from one of the two sets of lacustrine argillaceous source rocks present in the Pre-salt section of the Santos Basin, namely the Itapema Formation and the Picarras Formation.

The geochemical characteristics of organic matter in source rocks often exhibit similarities with the oil and gas generated from these source rocks^37,53,54^. Therefore, establishing a corresponding relationship between crude oil and its source rock can be achieved by comparing geochemical characteristic parameters. It’s worth noting that the structure of biomarkers is generally stable, especially at a lower maturity degree, where biological structures undergo relatively little change. Therefore, the structural characteristics of biomarkers of the Barra Velha Formation crude oil and Itapema Formation source rock can be compared to identify the source of Barra Velha Formation crude oil. If the matching degree between the two is good, it can be considered that the crude oil of the Barra Velha Formation comes from the source rock of the Itapema Formation. If the matching degree is poor, it can be considered that the source rock is the Picarras Formation.

The n-alkanes of crude oil within the Barra Velha Formation in the study area indicate that its organic matter origin is low aquatic plants, and the regular steranes of the source rocks of the Itapema Formation are dominated by C_27_ steranes, indicating that the source of organic matter is mainly algae or some other low aquatic organisms (Figs. 5 and 9). In addition, the crude oil in the study area has the characteristics of Ts < Tm and a high content of gammacerane. It is speculated that the parent material is derived from the sedimentary environment of the salt lake, and the source rock of Itapema Formation also shows the same sedimentary environment characteristics of the salt lake (Figs. 4 and 8). The saturated hydrocarbon C_29_ sterane 20S/(20R + 20S) ratio of crude oil in the study area is between 0.372 and 0.51, indicating that the maturity of crude oil is in the middle-high maturity stage, which is also consistent with the source rock of Itapema Formation (Figs. 5 and 9). Based on the above analysis, it is considered that the crude oil of Barra Velha Formation in the study area comes from the source rock of Itapema Formation.

### Oil and gas charging period and direction

#### Oil and gas accumulation process in the STS-C block

The STS-C block is located in the Lula-Sugar uplift belt (Fig. 1), with a water depth of about 2000–2200 m. It is dominated by medium crude, with a small amount of H_2_S, CO_2_ content of about 45.00 mol%, and gas-oil ratio between 326.00 m^3^/m^3^ and 489.12 m^3^/m^3^. To validate the accuracy of the hydrocarbon charging period derived from the source–reservoir dynamic evaluation method, an analysis of hydrocarbon inclusions within the Barra Velha Formation of the STS-64 well, situated in the STS-C block within the Santos Basin, was conducted as an initial step.

**Figure 10:**
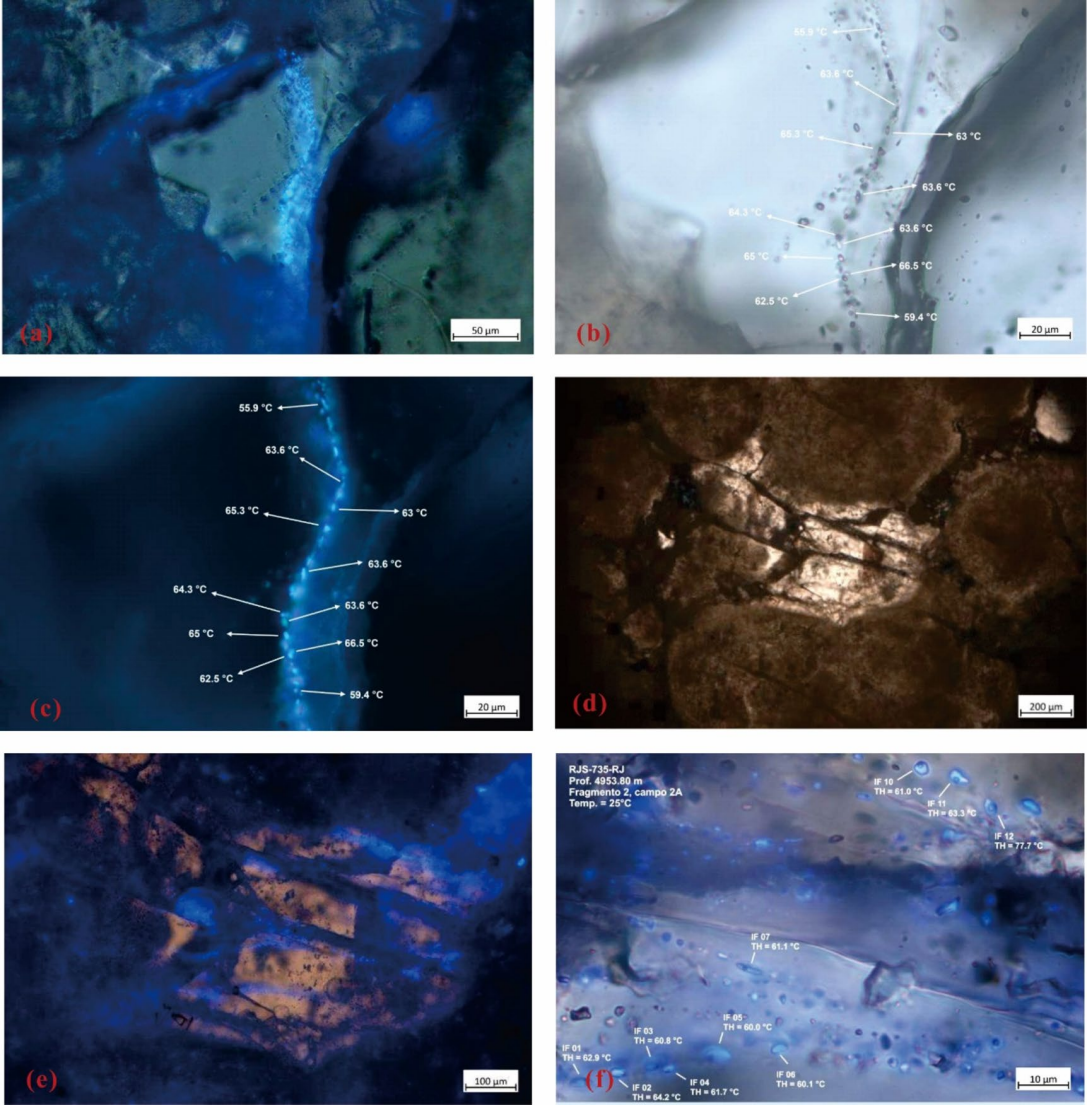
Fluid inclusion characteristics of Barra Velha Formation in Well STS-64, Block STS-C, Santos Basin, the results of sampling depth 4949.90 m are: (**a**) hydrocarbon inclusion microscopic fluorescence photographs, (**b**) locally amplified transmitted light photographs and (**c**) locally amplified microscopic fluorescence photographs and temperature measurement results; The results at a sampling depth of 4953.80 m are: (**d**) transmitted light photographs of hydrocarbon inclusions, (**e**) microscopic fluorescence photographs and (**f**) locally amplified microscopic fluorescence photographs and temperature measurement results.

**Figure 11:**
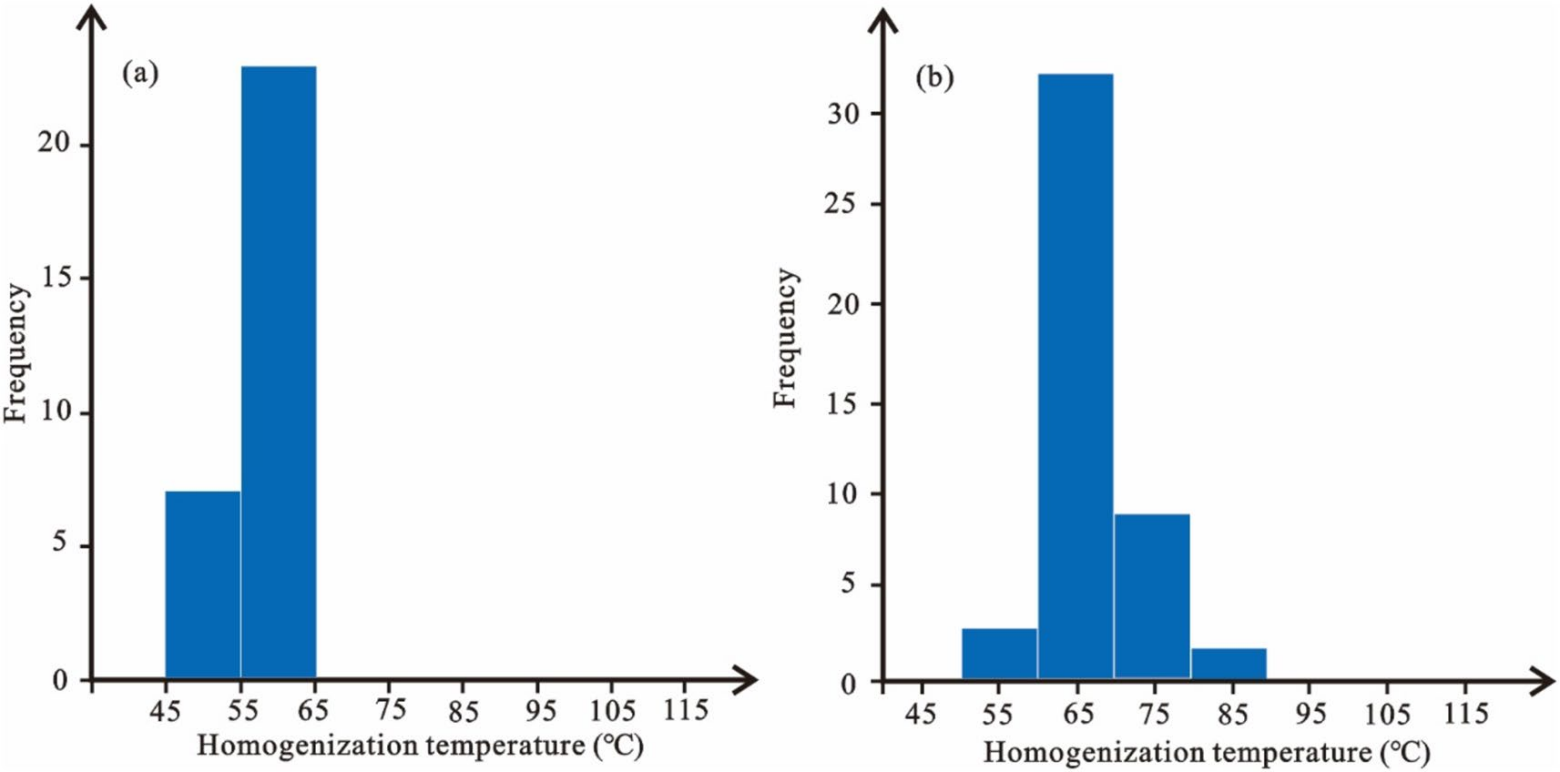
Homogenization temperature of fluid inclusion in Barra Velha Formation, Well STS-64, Block STS-C, Santos Basin, (**a**) hydrocarbon inclusion at a sampling depth of 4949.90 m, and (**b**) hydrocarbon inclusion at a sampling depth of 4953.80 m.

The examination results reveal that hydrocarbon inclusions are primarily located within healing cracks of barite and fractures traversing calcite. They are uneven in size, often distributed in groups, with different shapes, mostly irregular shapes, and long strips and ovals are only occasionally visible. Under the transmitted light, hydrocarbon inclusions are mostly transparent and colorless, and a small amount is brown or light brown, with obvious black bubble edges. Under microscopic fluorescence, the hydrocarbon inclusions are mainly blue-white, indicating that the maturity of the oil is middle to high (Fig. 10). The homogenization temperature of fluid inclusions in samples obtained at a depth of 4949.90 m falls within the range of 45–65 °C, with the majority clustered between 55 and 65 °C. Similarly, samples retrieved at a depth of 4953.80 m exhibit homogenization temperatures spanning from 50 to 90 °C, mainly 60–70 °C (Fig. 11).

**Figure 12:**
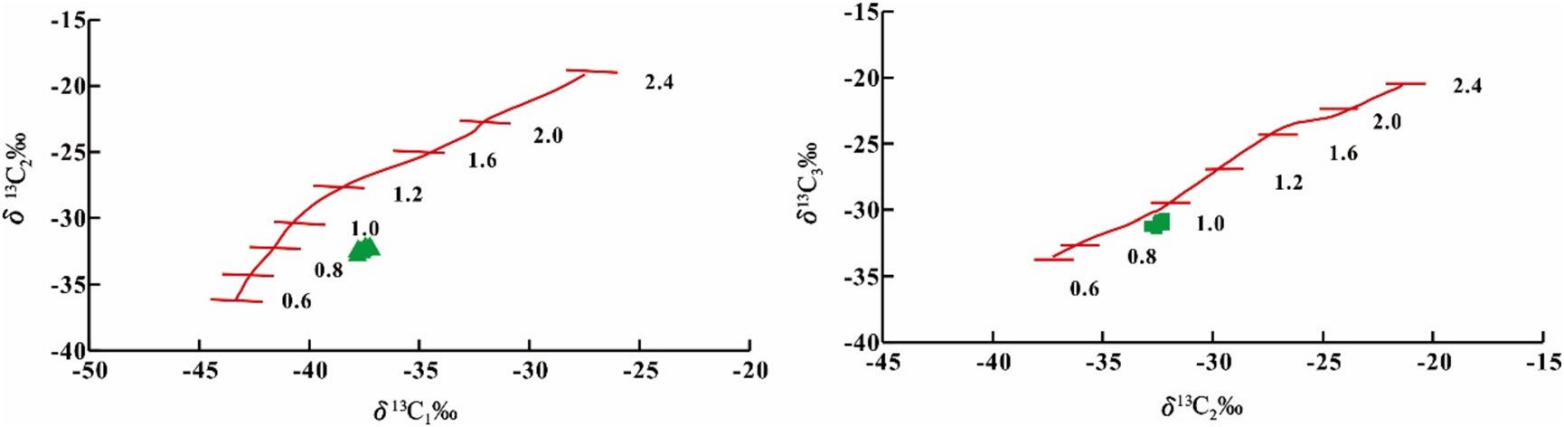
The relationship between natural gas (δ^13^C_1_–δ^13^C_2_), (δ^13^C_2_–δ^13^C_3_) of Barra Velha Formation in Wells STS-67 and STS-92 and maturity of source rock, Block STS-C, Santos Basin^51,52^.

The correlation between the biomarker compound parameters of the crude oil and source rock maturity within the STS-C block suggests that the crude oil within this region originates from source rocks with a maturity level ranging from 0.60 to 0.80% (Tables 1 and 2). Furthermore, an examination of the carbon isotope characteristics of natural gas, (δ^13^C_1_–δ^13^C_2_) and (δ^13^C_2_–δ^13^C_3_), indicate that the natural gas is mainly the product of source rocks at a maturity of 0.8–1.0% (Fig. 12). Through a comprehensive analysis, it becomes evident that the oil and gas reservoirs within the STS-R block primarily originate from source rocks with a maturity level spanning from 0.6 to 1.0%.

**Table 1:** The correlation between the biomarker compound parameters of the crude oil and source rock maturity.

Biomarker compound parameters	R_o_ = 0.6%	R_o_ = 0.8%	1.1% < R_o_ < 1.3%	R_o_ = 1.4%	References
C_29_20S/(20R + 20S)	0.25	0.4	0.50–0.55	/	^55–57^
Ts/(Ts + Tm)	/	0.45	0.80–0.85	1	^56^

**Table 2:** Crude oil maturity of Barra Velha formation in STS-C block and corresponding source rock maturity.

Well No.	Ts/(Ts + Tm)	C_29_20S/(20R + 20S)	Maturity of Source Rock
STS-92	0.245	0.451	0.6% < R_o_ < 0.8%
	0.248	0.437	0.6% < R_o_ < 0.8%
	0.245	0.444	0.6% < R_o_ < 0.8%
	0.255	0.450	0.6% < R_o_ < 0.8%
	0.248	0.438	0.6% < R_o_ < 0.8%
	0.246	0.423	0.6% < R_o_ < 0.8%
	0.245	0.416	0.6% < R_o_ < 0.8%
**STS-67**	0.242	0.451	0.6% < R_o_ < 0.8%
	0.240	0.434	0.6% < R_o_ < 0.8%

**Figure 13:**
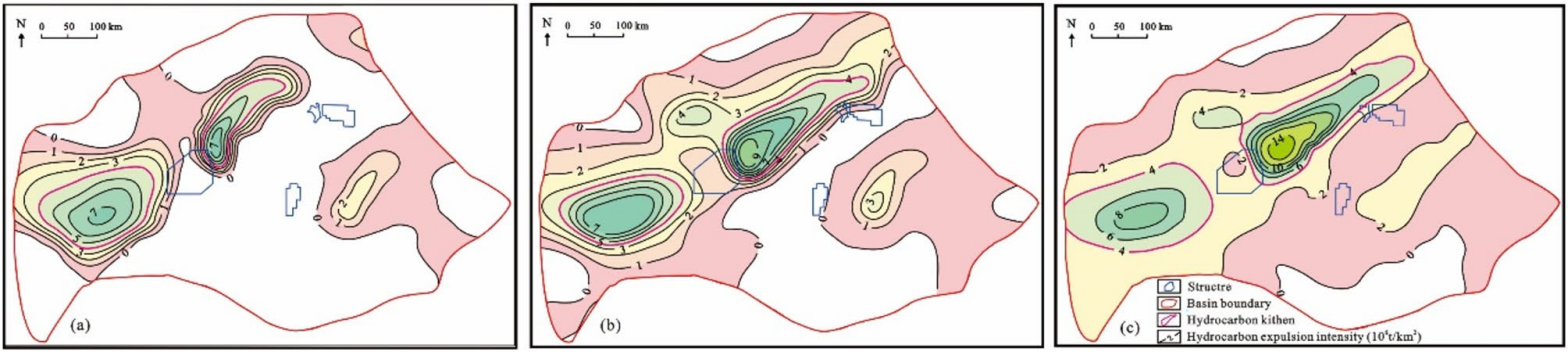
The plane distribution of hydrocarbon expulsion characteristics and the range of source kitchen in key geological periods of Itapema Formation, Santos Basin, (**a**) the Late sedimentary period of Florinopolis Formation (95 Ma), (**b**) the Late sedimentary period of Santos Formation (95 Ma), (**c**) the present day.

**Figure 14:**
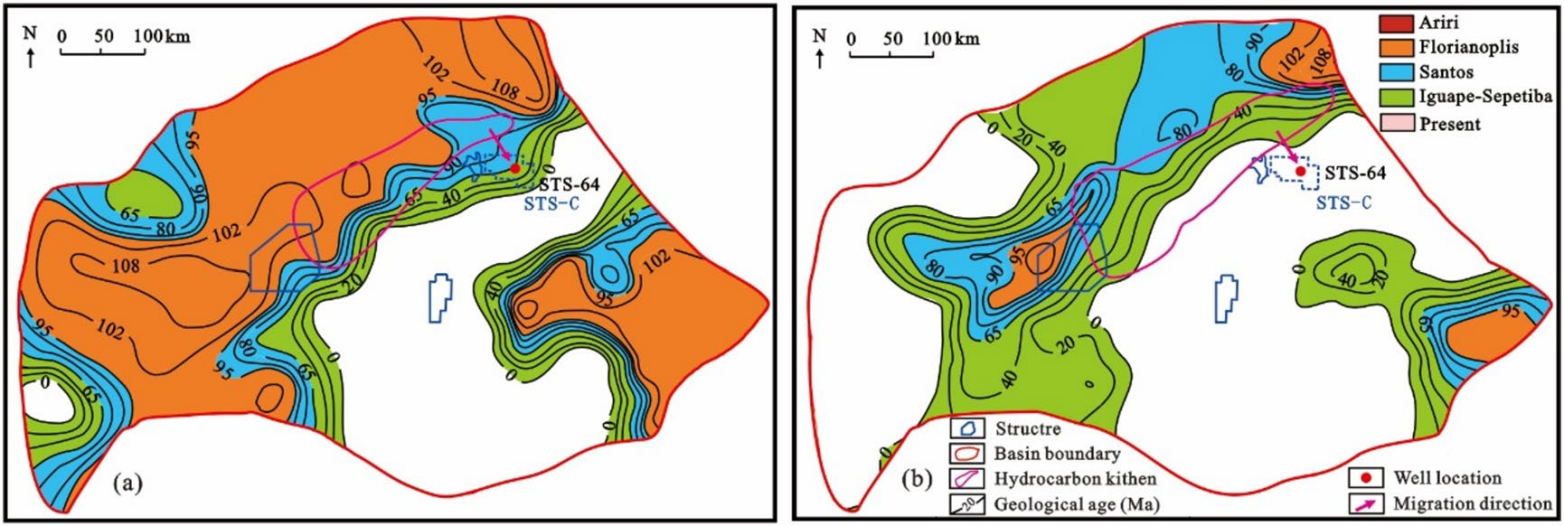
The geological time plane distribution map of the bottom of the source rock at maturity of 0.6% (**a**) and the top of the source rock at maturity of 1.0% (**b**) of Itapema Formation in Santos Basin, and the range of source kitchen in corresponding geological periods.

According to the characteristics of hydrocarbon expulsion history of source rocks in the Itapema Formation, the range of source kitchen in key geological periods is delineated (Fig. 13). The geological time plane distribution characteristics when the bottom of the Itapema Formation begins to reach the lower limit of oil and gas maturity (0.6%) in the STS-C block, and the top of the Itapema Formation begins to reach the upper limit of oil and gas maturity (1.0%) in the STS-C block are restored (Fig. 14).

The range of the source kitchen is superimposed to the geological time plane distribution maps where the bottom of the Itapema group begins to reach 0.6% and the top begins to reach 1.0%. Then the geological time period within the range of the source kitchen is the possible charging period of oil and gas: The period o during which oil and gas charging took place in the block STS-C spans from the Early deposition period of the Florianopolis Formation to the present day (108–0 Ma) (Fig. 14).

Based on the tectonic evolution of the basin and the vitrinite reflectance of the source rocks, the tectonic-thermal history of the Santos Basin is restored. To provide further clarity on the main charging period, a virtual well, designated as VW-1, was established within the source kitchen to simulate the thermal evolution history and hydrocarbon generation and expulsion history of the source rock according to the tectonic-thermal history. The results show that the source kitchen of the Itapema Formation reached to the low maturity (R_o_ = 0.5%), medium maturity (R_o_ = 0.7%), high maturity (R_o_ = 1.0%) and wet gas generation stage (R_o_ = 1.3%) at 108 Ma, 85 Ma, 62 Ma and 30 Ma, respectively. Notably, the source rock has not yet reached the dry gas generation stage, which determines that this block is oil reservoir, the API (American Petroleum Institute) gravity is relatively low, and the gas-oil ratio is relatively low. With the rapid increase of burial depth, the thermal evolution degree of source rock increases rapidly, and the main oil generation period is from the deposition period of the Santos Formation to the deposition period of the Iguape Formation (85–58 Ma). With the oil is generated in large quantities, it is expelled from the source rock, and the main oil expulsion period is from the deposition period of the Santos Formation to the deposition period of the Iguape Formation (80–62 Ma).

**Figure 15:**
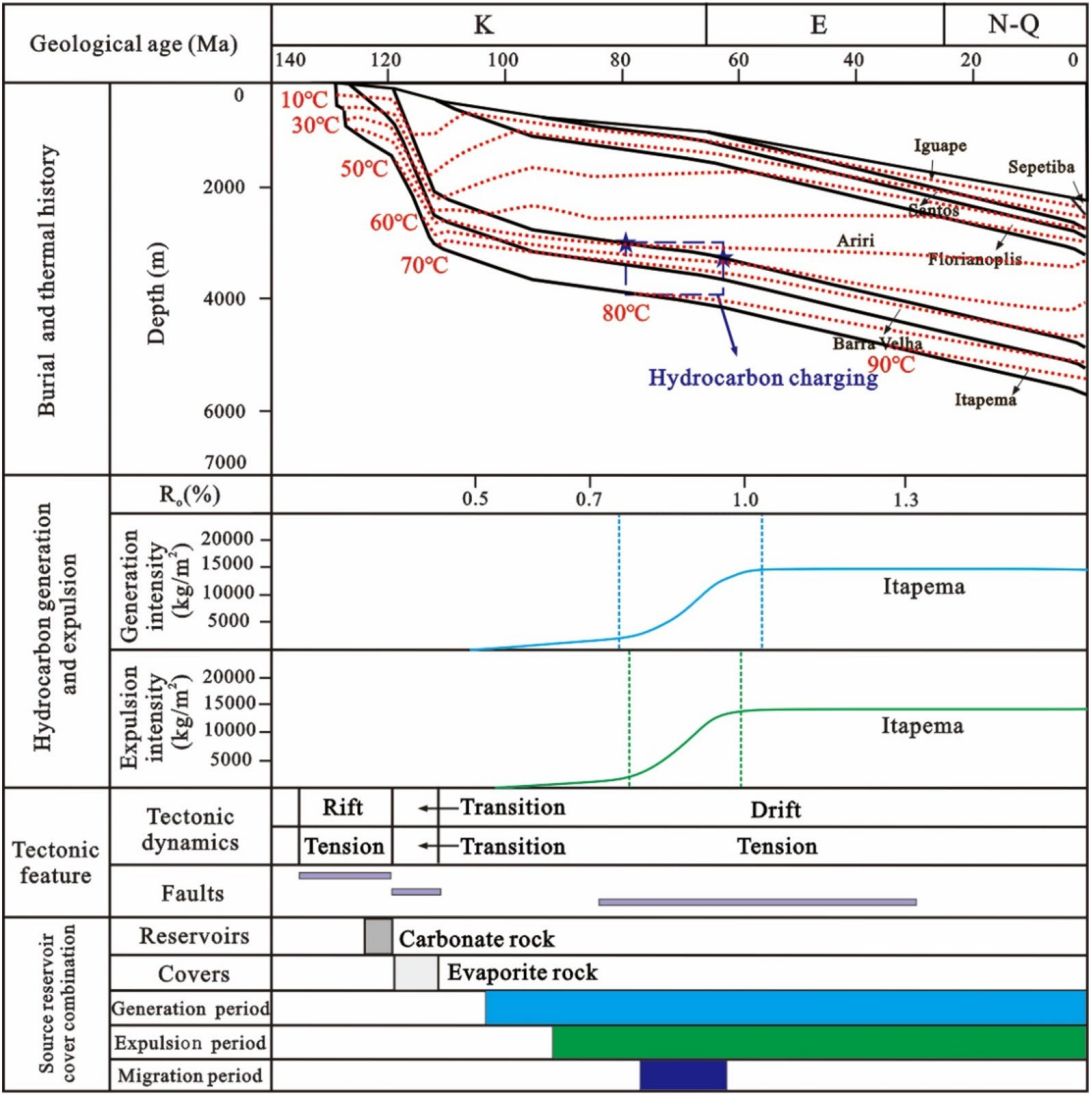
Reservoir formation process diagram of well STS-64, Block STS-C, Santos Basin.

Building upon these findings, the accumulation process of the STS-64 well can be analyzed as follows: The reservoir within the STS-C block primarily consists of carbonate rock from the Barra Velha Formation, while the caprock mainly comprises evaporate salt rock from the Ariri Formation. Migration pathways are predominantly defined by faults originating from the rift and transitional stages. The source rock has a large amount of tectonic subsidence and a large burial depth in the geological history period. It enters the hydrocarbon generation and expulsion stage very early, and the accumulation period is also very early. The main accumulation period is in the deposition period of the Santos Formation (80–65 Ma) (Fig. 15).

Given the close proximity of the depths at which the two samples were obtained, the homogenization temperatures of the fluid inclusions from both samples can be combined for analysis. The results show that the capture temperature is between 50 and 90 °C, with a significant frequency (64%) concentrated in the 60–70 °C, which is the main capture temperature range (Fig. 15). This temperature range is then overlaid onto the burial and thermal history map of a single well, leading to the determination of the hydrocarbon charging period, which spans from 80 to 68 Ma. The high degree of alignment between the primary charging period identified through traditional fluid inclusion analysis and the period determined via the assessment of thermal evolution history of source rocks and the maturity of crude oil and natural gas provides compelling evidence for the reliability of the new method introduced in this paper.

#### Oil and gas accumulation process in the STS-R block

**Figure 16:**
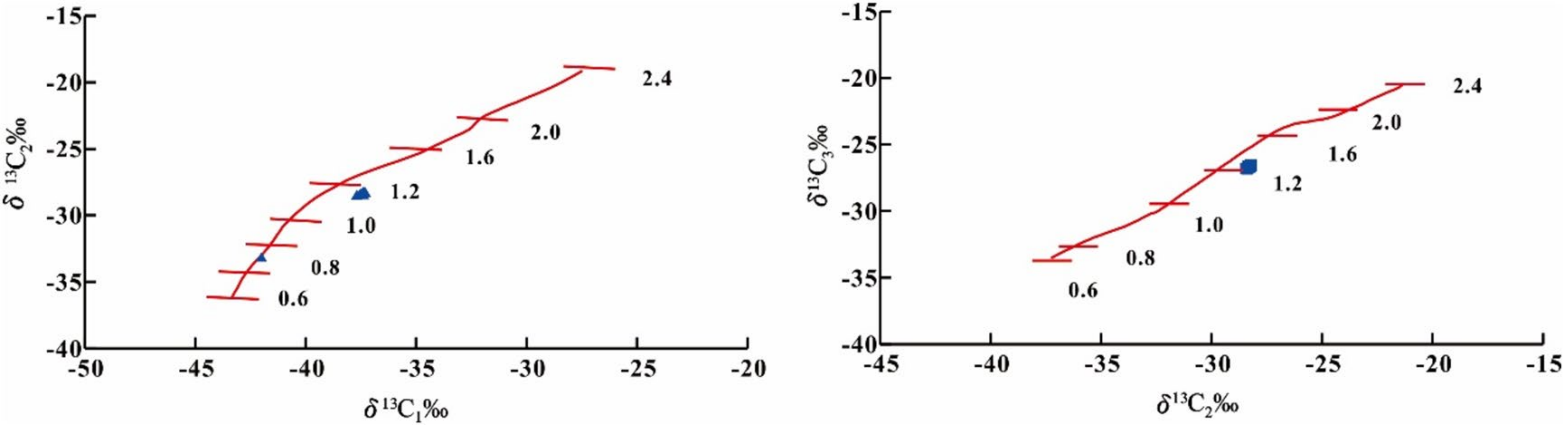
The relationship between natural gas (δ^13^C_1_–δ^13^C_2_), (δ^13^C_2_–δ^13^C_3_) of Barra Velha Formation in Well 665 and maturity of source rock, Block STS-R, Santos Basin.

The STS-R block is located in the middle of the sea area of the Santos Basin and structurally located in the Aram-Uirapuru uplift belt with a water depth of 300–2200 m (Fig. 1). It is one of the top ten crude oil discoveries in the world in the past ten years. It is mainly composed of medium crude, a small amount of H_2_S, CO_2_ content is about 4.57 mol%, and the gas-oil ratio is between 181.60 m^3^/m^3^ and 312.81 m^3^/m^3^. The Barra Velha Formation of the STS-665 well in the STS-R block has good oil and gas shows. The correlation established between the biomarker compound parameters of the crude oil and source rock maturity suggests that the crude oil within the STS-R block originates from source rocks with a maturity level spanning from 0.8 to 1.1% (Table 3). The carbon isotope characteristics of natural gas, (δ^13^C_1_–δ^13^C_2_) and (δ^13^C_2_–δ^13^C_3_), indicate that the natural gas is mainly the product of source rocks at a maturity of 1.0–1.2% (Fig. 16). Upon comprehensive analysis, it is apparent that the oil and gas reservoirs within the STS-R block primarily originate from source rocks with a maturity level spanning from 0.8 to 1.2%.

**Table 3:** Crude oil maturity of Barra Velha formation in STS-R block and corresponding source rock maturity.

Ts/(Ts + Tm)	C_29_20S/(20R + 20S)	Source rock maturity
0.438	0.429	0.8% < R_o_ < 1.1%
0.450	0.381	0.8% < R_o_ < 1.1%
0.454	0.387	0.8% < R_o_ < 1.1%
0.429	0.394	0.6% < R_o_ < 0.8%
0.443	0.402	0.8% < R_o_ < 1.1%
0.437	0.380	0.6% < R_o_ < 0.8%
0.436	0.379	0.6% < R_o_ < 0.8%
0.498	0.399	0.8% < R_o_ < 1.1%
0.498	0.372	0.8% < R_o_ < 1.1%
0.490	0.393	0.8% < R_o_ < 1.1%

**Figure 17:**
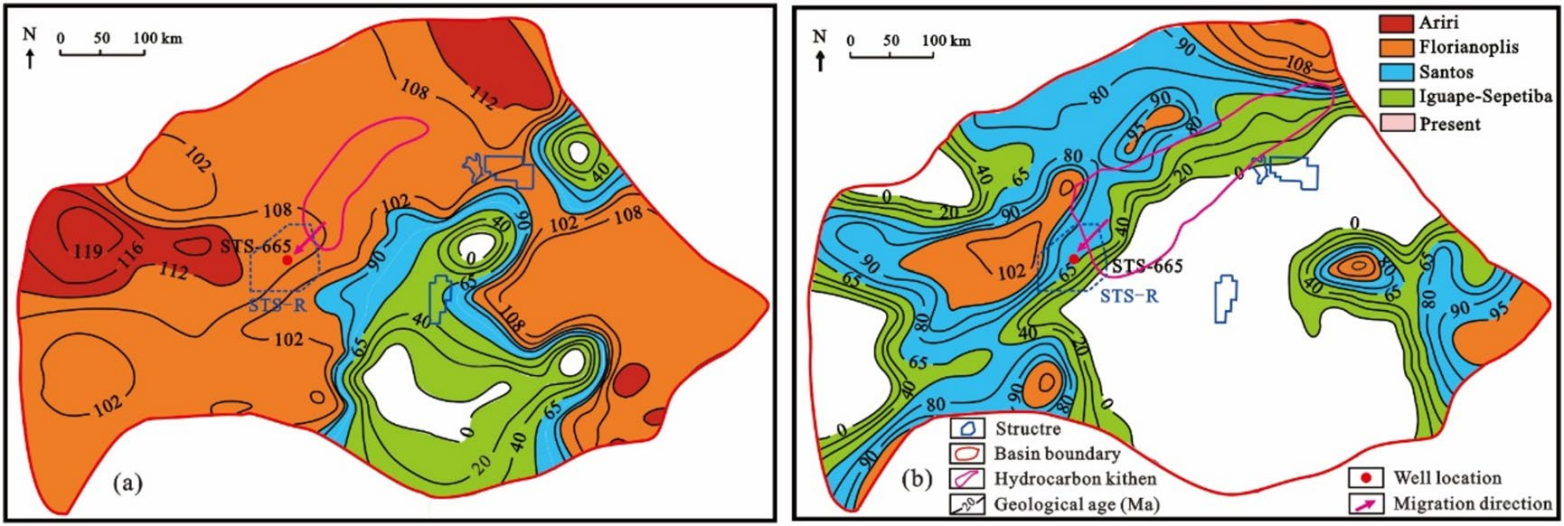
The geological time plane distribution map of the bottom of the source rock at maturity of 0.8% (**a**) and the top of the source rock at maturity of 1.2% (**b**) of Itapema Formation in Santos Basin, and the range of source kitchen in corresponding geological periods.

The geological time plane distribution characteristics when the bottom of the source rock of the Itapema Formation began to reach the lower limit of oil and gas maturity (the source rock at maturity of 0.8%), and when the top of the source rock of the Itapema Formation began to reach the upper limit of oil and gas maturity (the source rock at maturity of 1.2%) in the STS-R block were restored. It’s important to note that this analysis focuses exclusively on the Eastern Sag adjacent to the STS-R block, given the limited exploration in the Western Sag. The source kitchen ranges of the Florianopolis Formation sedimentary period and the current are superimposed on the geological time plane distribution map of the bottom of the source rock of the Itapema Formation starting to reach 0.80% and the top starting to reach 1.20%, respectively. The results show that the oil and gas charging time of the STS-R block is from the deposition period of the Florianopolis Formation to the nowadays (102–0 Ma) (Fig. 17).

The simulation results of thermal evolution history and hydrocarbon generation and expulsion history of source rocks in the well VW-2 show that the source kitchen of Itapema Formation reached the low maturity (0.5%) stage, medium maturity (0.7%) stage, and high maturity (1.0%) stage at 102 Ma, 84 Ma, and 16 Ma, respectively, and have not yet entered the gas generation stage currently. The principal period of hydrocarbon generation is identified as spanning from the sedimentary phase of the Florianopolis Formation to the Santos Formation (80–40 Ma), and the main oil expulsion period is from the Florianopolis Formation sedimentary period to the Santos Formation sedimentary period (78–45 Ma).

**Figure 18:**
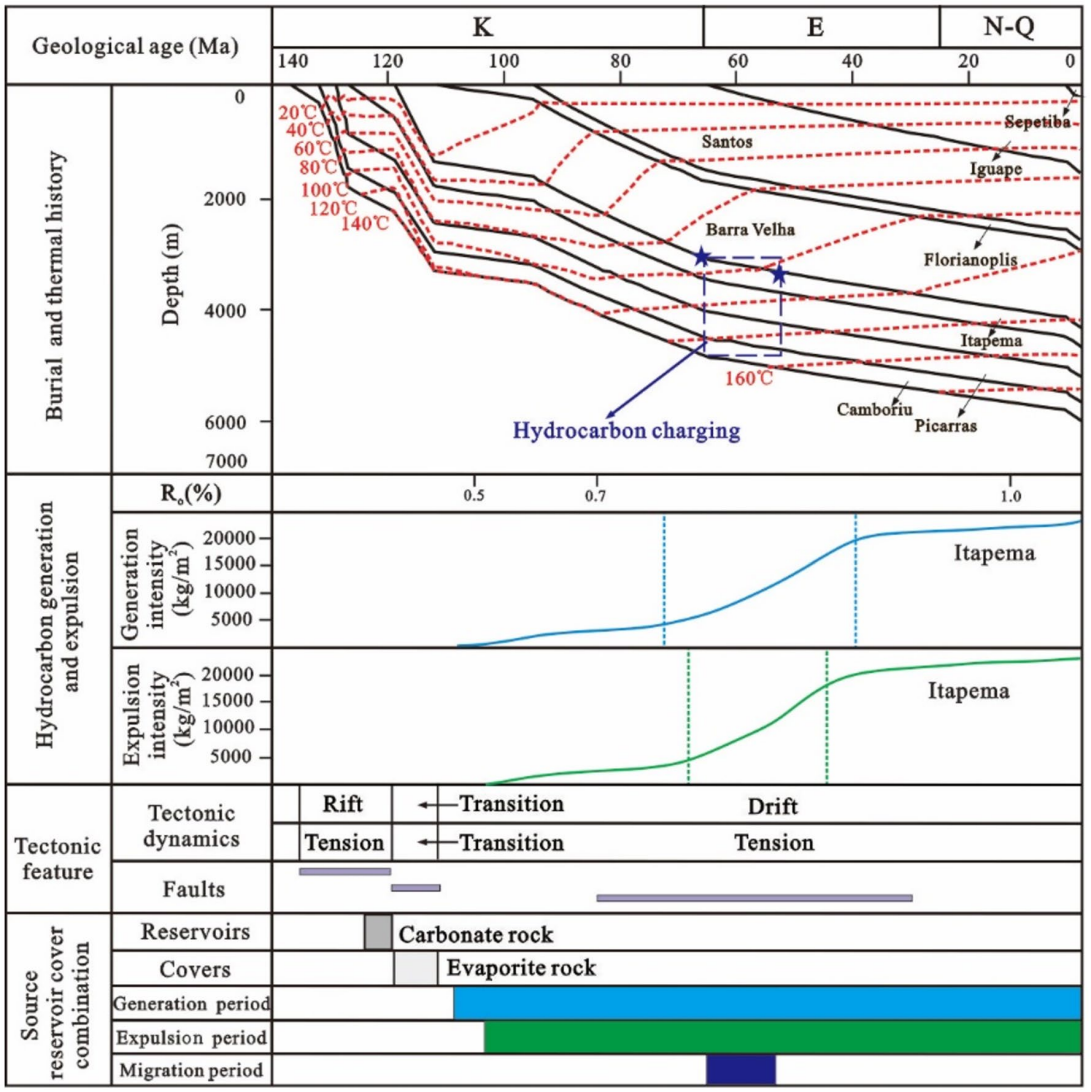
Reservoir formation process diagram of well STS-665, Block STS-R, Santos Basin.

Combined with the above results, the accumulation process of the STS-665 well was analyzed (Fig. 18): The reservoir of the STS-R block is mainly carbonate rock of the Barra Velha Formation, the caprock is mainly the Ariri Formation evaporate salt rock, and the migration channels are mainly faults formed in the rift stage and the transitional stage. The main accumulation period is from the Late Santos Formation sedimentary period to the Early Iguape Formation sedimentary period (65–55 Ma).

#### Oil and gas accumulation process in the STS-A block

The STS-A block is located in the Lula-Sugar uplift belt, adjacent to the eastern sag of the central depression zone (Fig. 1). It is mainly composed of medium crude, CO_2_ content is between 8.00 and 18.00 mol%, and the gas-oil ratio is between 269.60 and 282.00 m^3^/m^3^. Through the conversion relationship between the methylphenanthrene index (MPI) of crude oil and the vitrinite reflectance of the source rock, it is clear that the crude oil in the STS-A block is the product of the source rock at the maturity of 0.83–0.90% (Table 4).

**Table 4:** Crude oil maturity in Block STS-A, Santos Basin.

Well No.	MPI	Rc(0.65% < Rm < 1.35%)
STS-1	0.83	0.90
STS-272	0.72	0.83

**Figure 19:**
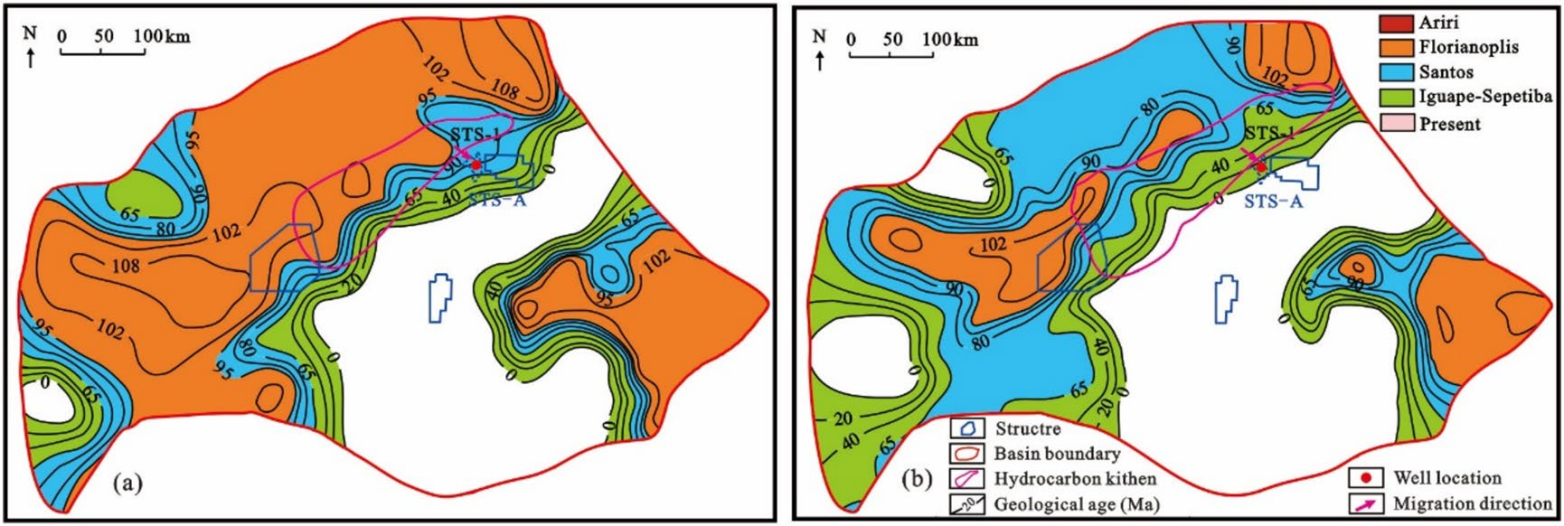
The geological time plane distribution map of the bottom of the source rock at maturity of 0.83% (**a**) and the top of the source rock at maturity of 0.90% (**b**) of Itapema Formation in Santos Basin, and the range of source kitchen in corresponding geological periods.

The geological time plane distribution characteristics when the bottom of the source rock of the Itapema Formation began to reach the lower limit of oil and gas maturity (the source rock at maturity of 0.83%), and when the top of the source rock of the Itapema Formation began to reach the upper limit of oil and gas maturity (the source rock at maturity of 0.90%) in the STS-A block were restored. The source kitchen ranges of the Florianopolis Formation sedimentary period and the current are superimposed on the geological time plane distribution map of the bottom of the source rock of the Itapema Formation starting to reach 0.83% and the top starting to reach 0.90%, respectively. The results show that the oil and gas charging period of the STS-A block is from the Middle Florianopolis Formation sedimentary period to the nowadays (98–0 Ma) (Fig. 19).

**Figure 20:**
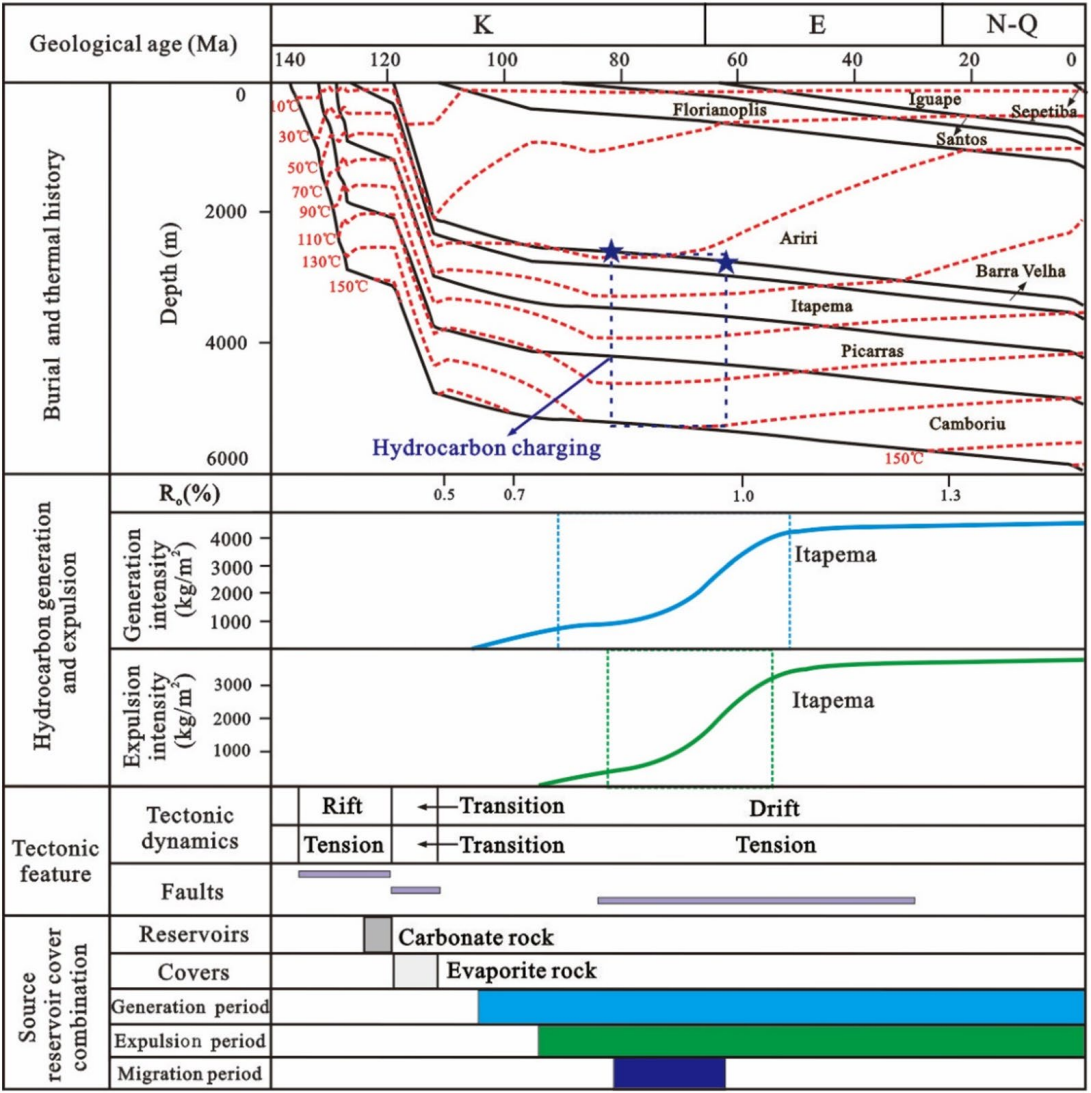
Reservoir formation process diagram of well STS-1, Block STS-A, Santos Basin.

The simulation results of thermal evolution history and hydrocarbon generation and expulsion history of source rocks in the VW-3 well show that the source kitchen of Itapema Formation reached the low maturity (0.5%) stage, medium maturity (0.7%) stage, high maturity (1.0%) stage and wet gas generation stage (1.3%) at 110 Ma, 101 Ma, 60 Ma, and 24 Ma, respectively, and have not entered the dry gas generation stage currently. The main oil generation period is from the Florianopolis Formation sedimentary period to the Santos Formation sedimentary period (92–54 Ma), and the main oil expulsion period is from the Florianopolis Formation sedimentary period to the Santos Formation sedimentary period (83–58 Ma). The main accumulation period was during the Santos Formation sedimentary period (82–65 Ma). Combined with the above results, the accumulation process of the STS-1 well is analyzed (Fig. 20): The reservoir of the STS-A block is mainly carbonate rock of the Barra Velha Formation, the caprock is mainly the Ariri Formation evaporate salt rock, and the migration channels are mainly faults formed in the rift stage and the transitional stage. The main accumulation period is during the Santos Formation sedimentary period (82–65 Ma).

#### Oil and gas accumulation process in the STS-Q block

The STS-Q block is located in the Eastern Depression zone (Fig. 1). The main production layers are the carbonate rocks of the Barra Velha Formation and the Itapema Formation. Only a very thick high-CO_2_ condensate gas layer is developed, and its CO_2_ content is as high as 95.5 mol%. There is no oil ring that can be seen or no oil ring at all.

**Figure 21:**
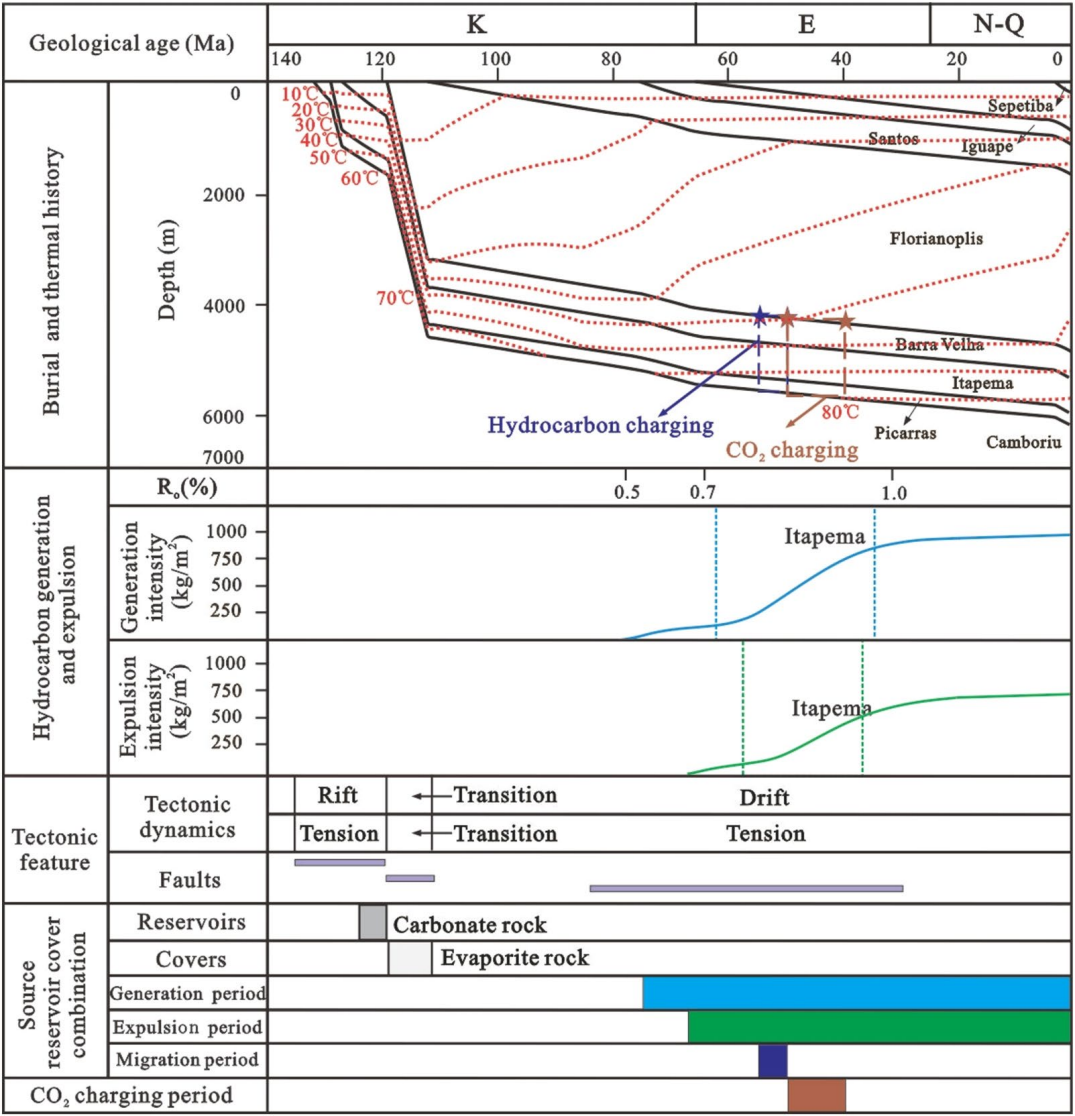
Reservoir formation process diagram of well STS-664, Block STS-Q, Santos Basin.

The northwestern side of the STS-Q block is the Central Depression zone, but there is a saddle between the Sugar Loaf sub-high and the STS-Q structure under the current salt bottom. Therefore, the STS-Q block is not the dominant migration direction for oil and gas generated in the Central Depression zone. A small depression has developed in the southeastern part of the STS-Q block, with a source rock development period of 129–127 Ma, a gas generation period from 78 Ma to the nowadays, a gas expulsion period of 68 Ma to the present day, and a main expulsion period of 55–50 Ma. However, the expulsion scale is very limited, with a maximum of only 745 kg/m^2^. Therefore, although the STS-Q block has been in the high part since the Middle deposition of the Florianopolis Formation (95–0 Ma), and has developed good anticline traps, which is conducive to oil and gas accumulation, the oil and gas charging is insufficient, and its accumulation scale is very limited (Fig. 21)^12,58^.

## Discussions

### Pre-salt hydrocarbon accumulation models

**Figure 22:**
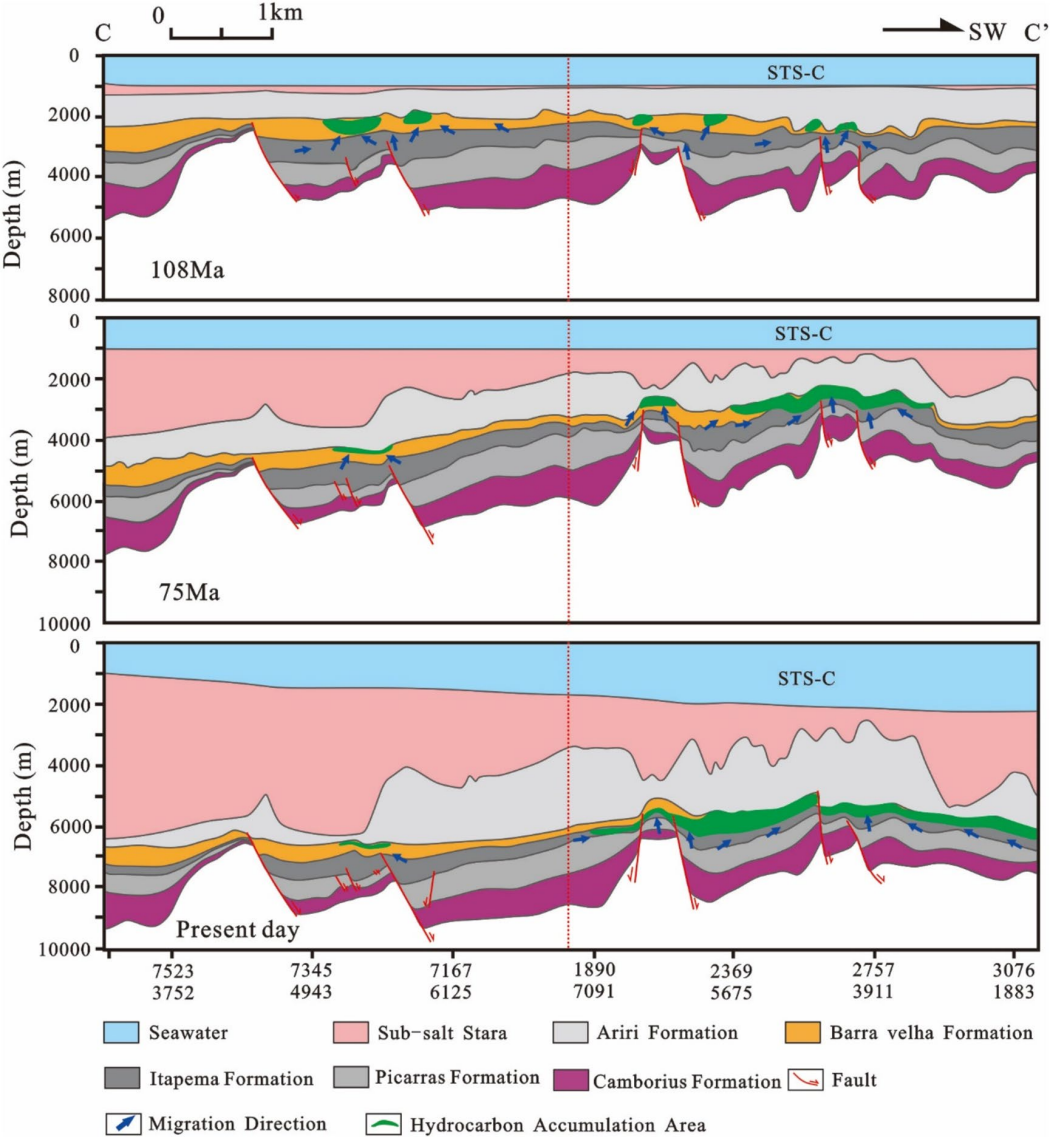
Schematic diagram of dynamic accumulation model in STS-C block, Santos Basin.

**Figure 23:**
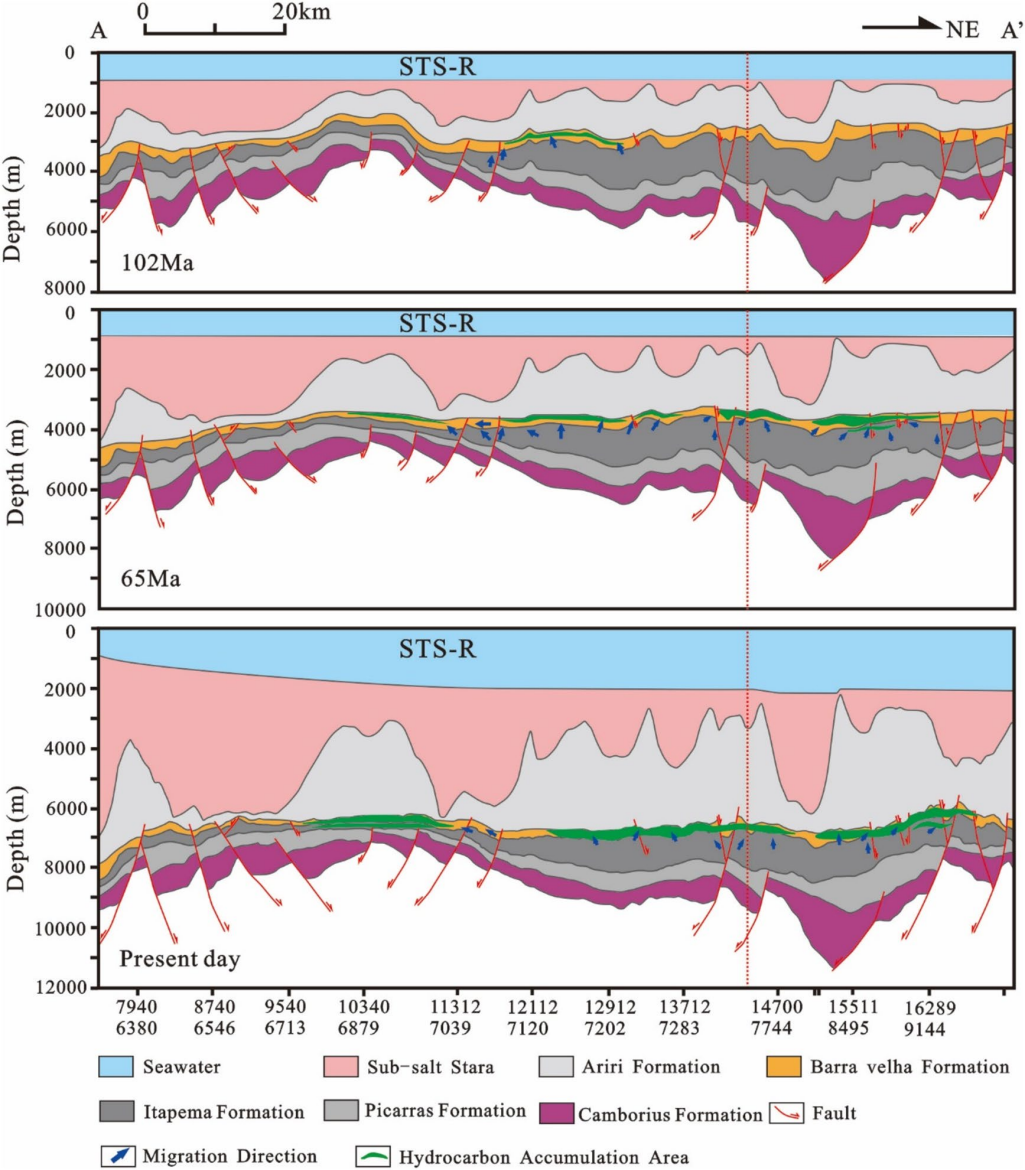
Schematic diagram of dynamic accumulation model in STS-R block, Santos Basin.

**Figure 24:**
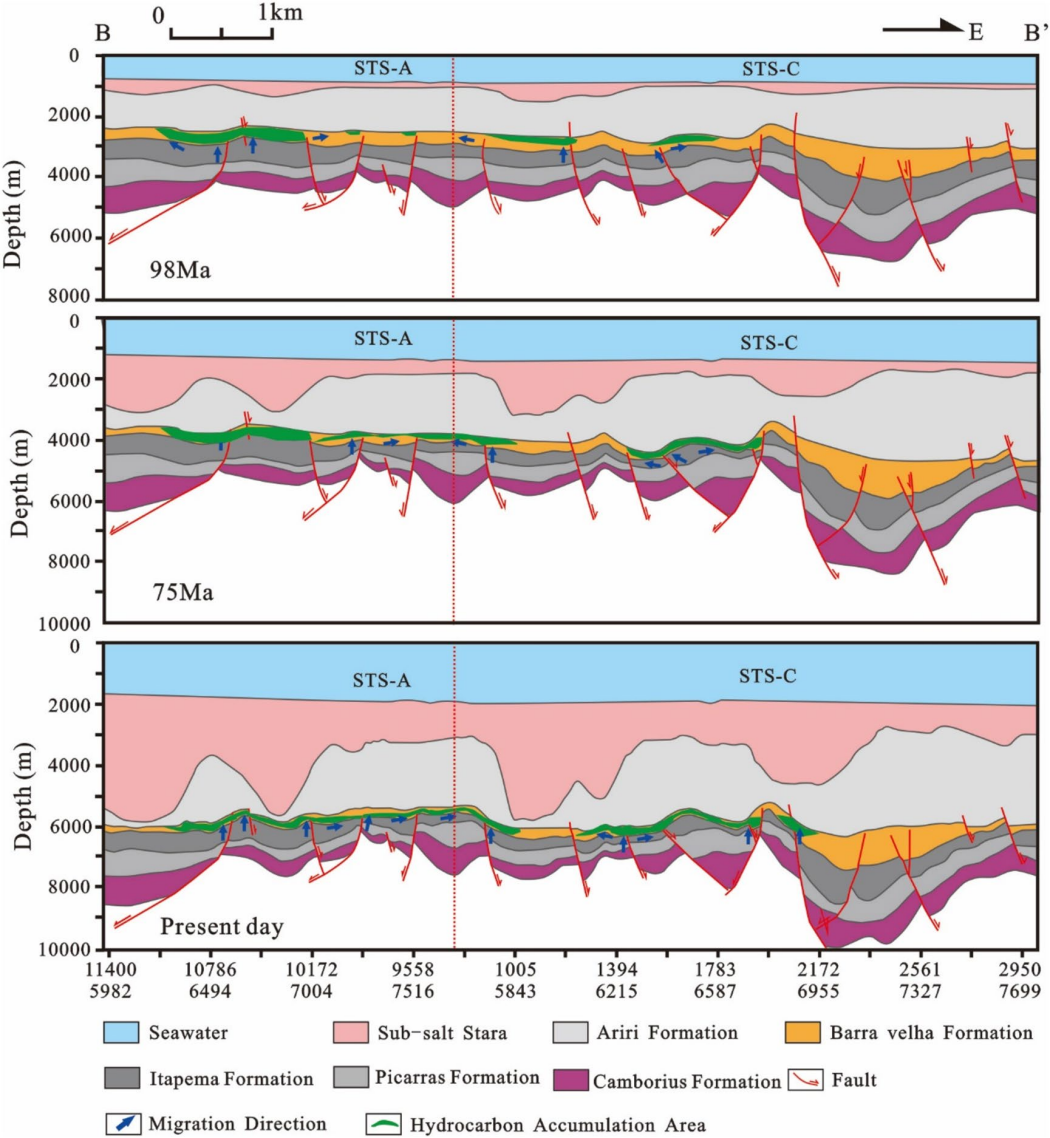
Schematic diagram of dynamic accumulation model in STS-A block, Santos Basin.

**Figure 25:**
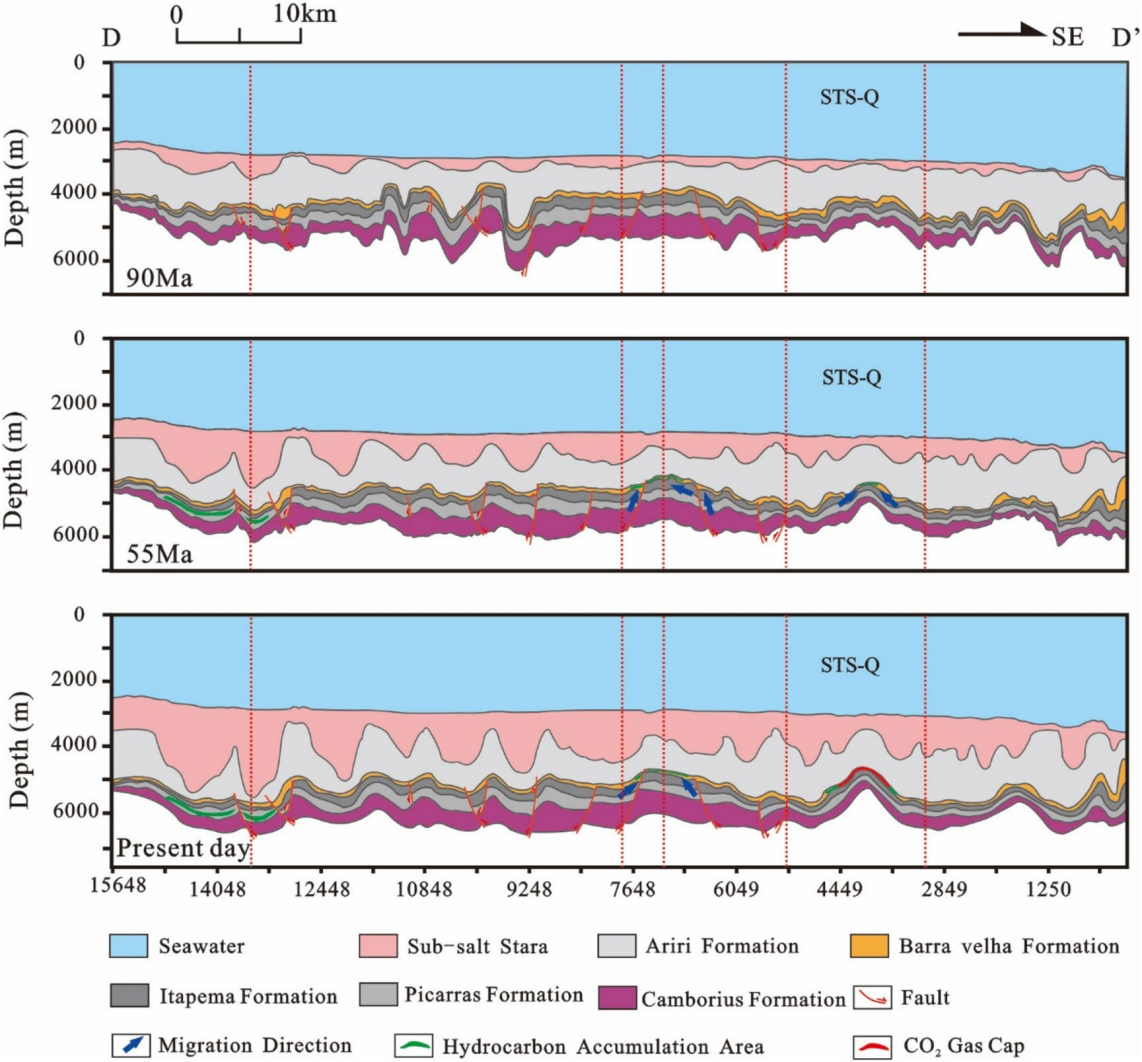
Schematic diagram of dynamic accumulation model in STS-Q block, Santos Basin.

The STS-C block, STS-R block, STS-A block, and STS-Q block are all elevated regions formed as a result of tectonic activity during the rift stage, which are conducive to the development of carbonate reservoirs. A significant feature in these areas is the presence of a thick, continuous salt layer overlay, which forms an anticline trap in conjunction with the underlying carbonate reservoirs. During the rift stage, extensive tectonic activity created numerous fault systems, serving as essential conduits for the vertical migration of oil and gas to these reservoirs. Consequently, oil and gas generated from the source rocks of the Itapema Formation migrated along these fault networks and accumulated within the carbonate reservoirs of the Barra Velha Formation (Fig. 22, 23, 24, 25).

Among them, the STS-R block is close to the eastern and western hydrocarbon generation sag (Fig. 23), the STS-C block (Fig. 22) and the STS-A block (Fig. 24) are all close to the main hydrocarbon generation sags located in the east side, and the hydrocarbon source conditions are good. Under the influence of fluid potential, oil and gas migrate to the high part of the structure in the permeable reservoir and accumulate, and the subsequent tectonic evolution also didn’t cause damage or transformation to these accumulations. In contrast, there is only a small source kitchen in the vicinity of the STS-Q block. Since 55 Ma, a small amount of hydrocarbon has migrated to the high part of the structure and accumulated in the permeable reservoir due to the influence of fluid potential, but the scale of accumulation is small. From 50 to 40 Ma, the mantle-derived CO_2_ gas in the basin enters the trap along the deep fault with magmatic activity, resulted a large amount of CO_2_ displaced oil and gas, forming a CO_2_ gas reservoir (Fig. 25).

**Figure 26:**
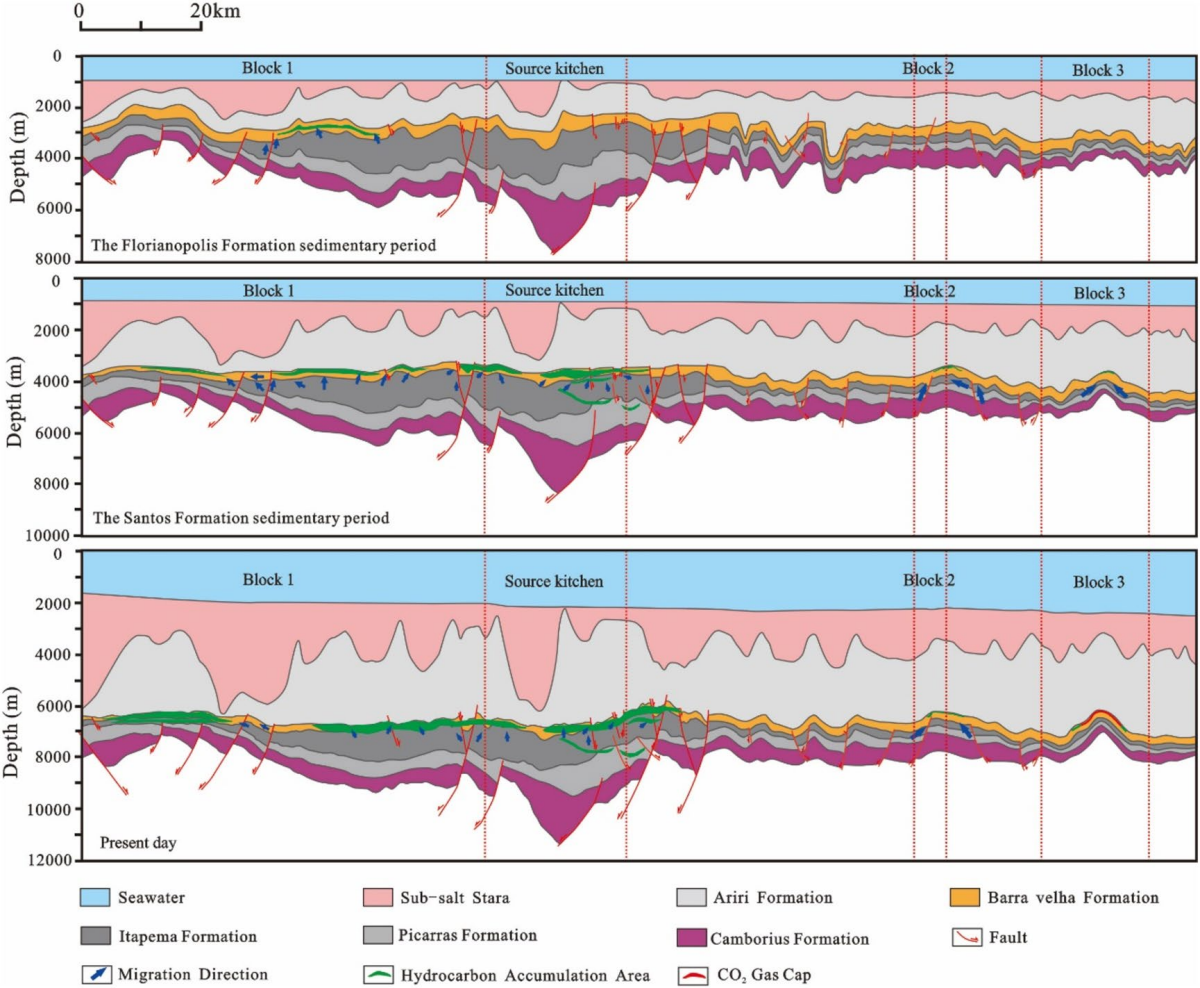
Conceptual dynamic accumulation model in Santos Basin. Hydrocarbon generated from source kitchen migrated along faults to adjacent fields to accumulate and have good oil and gas potential, such as block 1. Fields located far from source kitchen, such as block 2, have relatively poor oil and gas potential. In addition, some fields have poor oil and gas potential due to late CO_2_ charging, such as block 3.

Based on the analysis of the accumulation process and accumulation model of typical reservoirs, it is found that the pre-salt accumulation model of the Santos Basin is mainly the “lower generation-upper reservoir-salt cap” model (Fig. 26). In this model: The source rock is the lacustrine mudstone of the Itapema Formation. The main hydrocarbon generation period is from the Florianopolis Formation sedimentary period to the Santos Formation sedimentary period, the main hydrocarbon expulsion period is from the Santos Formation sedimentary period to the Early Iguape Formation sedimentary period, and the main hydrocarbon charging period is from the Late Santos Formation sedimentary period to the Early Iguape Formation sedimentary period. The carbonate rocks of the Barra Velha Formation and the overlying salt rocks of the Ariri Formation formed structural traps.

Hydrocarbons migrated vertically along the normal fault formed in the rift stage to the traps in the adjacent inherited tectonic highs, and accumulated in the traps, such as block 1. However, it is difficult for the oil and gas generated by source kitchen to migrate to the distant traps and accumulate. As a result, blocks located away from source rock ranges typically have lower hydrocarbon resource potential, such as Block 2. This accumulation model exhibits characteristics of being in proximity to the hydrocarbon source, often referred to as “near-source accumulation”. In addition, CO_2_ charging at a later stage will also lead to lower hydrocarbon resource potential, such as Block 3. Because the maturity of source rocks generally reaches the high maturity stage, with only a limited area reaching the gas generation stage, the reservoirs situated beneath the salt layers predominantly contain oil rather than natural gas.

### Petroleum exploration targets

The pre-salt hydrocarbon accumulation model in the Santos Basin indicates that the traps developed in the adjacent inherited tectonic high part adjacent to the source kitchen are favorable exploration areas for oil and gas. In order to quantitatively analyze the distribution probability of reservoirs and more accurately delineate favorable oil and gas exploration areas, the quantitative analysis model of the hydrocarbon distribution threshold proposed by Jiang et al. (2013) is introduced:

4 $$SI = 0.0{\text{46e}}^{{0.{12}q_{e}}} - 0.{\text{16ln}}L + 0.{\text{65e}}^{{ - {8}.{2357}\left( {{\text{l}} + 0.{1}} \right){2}}} + 0.{1345}$$

5 $$L = L_{i} /L_{0}$$

6 $$l= l_{i} /L_{0}$$

where, *SI* is the hydrocarbon accumulation probability; *q*_*e*_ is the maximum hydrocarbon discharge intensity of the hydrocarbon source rocks, 10^8^ t/km^2^; *L* is the standardized horizontal distance from the hydrocarbon discharge center to the oil and gas reservoir, dimensionless; *l* is the standardized horizontal distance from the hydrocarbon discharge boundary to the oil and gas reservoir, dimensionless; *L*_*i*_ is the actual horizontal distance from reservoir to hydrocarbon discharge center, km; *l*_*i*_ is the actual horizontal distance from reservoir to hydrocarbon boundary center, km; *L*_*0*_ is the horizontal distance from hydrocarbon discharge center to boundary along the direction of L, km.

This method holds that the number of the reservoirs and the distance from the center of hydrocarbon expulsion have a logarithmic decreasing trend, while the reservoirs at the boundary of hydrocarbon expulsion show the characteristics of concentrated distribution, which is characterized by Gaussian normal distribution. In addition, the number of reservoirs increases exponentially with the increase of hydrocarbon expulsion intensity, while due to the existence of a limit value of hydrocarbon expulsion intensity, there is an upper limit value of this number^59^.

**Figure 27:**
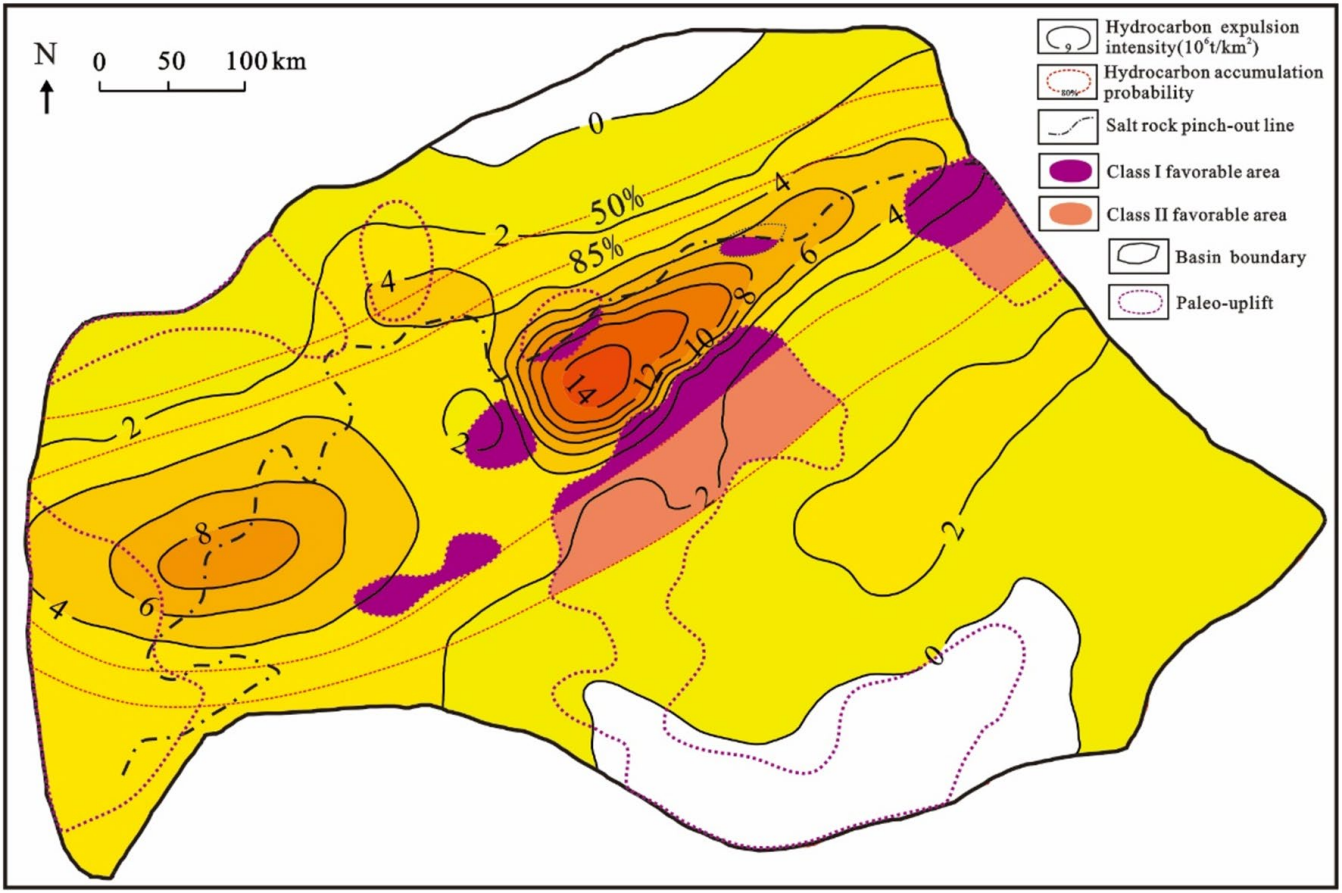
Optimal selection of pre-salt favorable area in Santos Basin.

Based on the hydrocarbon expulsion characteristics of source rocks within the Itapema Formation, this study employed the quantitative analysis model of the hydrocarbon distribution threshold to delineate two distinct accumulation probability ranges, namely 85% and 50% (Fig. 27). It is worth noting that while source rocks play a significant role in influencing hydrocarbon accumulation, other factors, particularly the ancient structural features of the main accumulation period, also exert a substantial influence. The most favorable areas for hydrocarbon accumulation are those that have experienced uplift from ancient times to the present, followed by regions that are presently elevated but were low-lying in ancient times. An important geological feature of the Santos Basin is the development of exceptionally thick salt rock layers during transitional periods. These salt rocks exhibit robust plasticity and serve as highly effective cap rocks for sealing hydrocarbons. Conversely, in the west-northwest margin of the basin, there is limited salt rock development, resulting in poorer sealing properties. Consequently, exploration efforts for pre-salt oil and gas reservoirs are more focused on areas with significant salt rock development.

Drawing upon the insights derived from source control theory and an understanding of ancient-to-modern structural patterns, the study concludes that the inherited structural highs and slopes falling within the 50% accumulation probability range represent favorable areas for future pre-salt oil and gas exploration efforts. Within this context, the ancient structural highs and slopes that lie within the 85% accumulation probability range are designated as Class I favorable areas, signifying areas with the highest potential for successful exploration. Conversely, ancient structural highs and slopes located within the 85% to 50% accumulation probability range are classified as Class II favorable areas, indicating areas with good exploration potential but somewhat lower than Class I areas It is suggested that the next oil and gas exploration should be carried out at the ancient structural highs and slopes of the favorable areas (Fig. 27).

## Conclusions

1. The crude oil of the Barra Velha Formation in the Santos Basin is dominated by medium crude, which is in the middle to high maturity stage. The drying coefficient of natural gas falling within the range of 0.78 to 0.91 indicates that the natural gas is characterized as “wet gas” and has not reached a high level of thermal maturity. The oil/gas-source rock correlation shows that the oil and gas in the reservoir of the Barra Velha Formation are mainly from the lacustrine argillaceous source rocks that belong to the Itapema Formation.
2. The main charging period of oil and gas in the STS-C block is determined according to the thermal evolution history of source rocks and the maturity of crude oil and natural gas. The results show that the STS-C block has been charged with oil and gas from the Eastern Sag since 108 Ma, and the charging intensity is largest from 80 to 65 Ma. The main charging period of oil and gas obtained by traditional fluid inclusions is from 80 to 68 Ma. The results obtained by the two methods have a high degree of matching, which proves that the new method proposed in this paper is reliable.
3. Using the source–reservoir dynamic evaluation technology, it is clear that the hydrocarbon charging period in the STS-R block is from the Florianopolis Formation sedimentary period to the present (102–0 Ma), and the main charging period is during the Late Santos Formation sedimentary period to the Early Iguape Formation sedimentary period (65–55 Ma). The hydrocarbon charging period of the STS-A block is from the Middle sedimentary period of the Florianopolis Formation to the present (98–0 Ma), and the Santos Formation sedimentary period (82–65 Ma) is the main charging period. The STS-Q block has been charged with hydrocarbon from a small sag in the southeast since 68 Ma, and the main charging period is the Early sedimentary period of the Iguape Formation (55–50 Ma).
4. The four blocks of STS-C, STS-R, STS-A, and STS-Q are all structural highs formed by tectonic activities in the rift stage. The carbonate reservoir of the Barra Velha Formation and the overlying Ariri Formation salt rock constitute anticline traps. A large number of fault-source faults formed by tectonic activities in the rift stage make oil and gas migrate to traps and accumulate. The three blocks of STS-C, STS-R, and STS-A are all close to the source kitchen, with sufficient hydrocarbon supply, large-scale oil and gas reservoirs, and good oil and gas shows. However, the hydrocarbon supply in the STS-Q block is insufficient, and the later CO_2_ charging leads to the displacement of hydrocarbon, thus forming a CO_2_ gas reservoir.
5. Based on the characteristics of “near-source accumulation” of pre-salt reservoirs in the Santos Basin, the quantitative analysis model of the hydrocarbon distribution threshold was introduced, and the distribution ranges of pre-salt accumulation probability of 85% and 50% in the Santos Basin were calculated respectively. It is suggested that the next oil and gas exploration should be carried out in the paleo-structural highs and slopes of Class I favorable area (accumulation probability is greater than 85%) and Class II favorable area (accumulation probability is 85–50%).

## Data Availability

Data is provided within the tables of the manuscript.
